# Utilization of a high-throughput shoot imaging system to examine the dynamic phenotypic responses of a C_4_ cereal crop plant to nitrogen and water deficiency over time

**DOI:** 10.1093/jxb/eru526

**Published:** 2015-02-19

**Authors:** E. H. Neilson, A. M. Edwards, C. K. Blomstedt, B. Berger, B. Lindberg Møller, R. M. Gleadow

**Affiliations:** ^1^School of Biological Sciences, Monash University, Clayton 3800, Australia; ^2^Plant Biochemistry Laboratory, Department of Plant and Environmental Sciences, University of Copenhagen, 40 Thorvaldsensvej, DK-1871 Frederiksberg C, Copenhagen, Denmark; ^3^The Plant Accelerator, Australian Plant Phenomics Facility, University of Adelaide, Glen Osmond 5064, Australia; ^4^Carlsberg Laboratory, Gamle Carlsberg Vej 10, DK-1799 Copenhagen V, Denmark

**Keywords:** Cyanogenesis, drought, growth, LemnaTec Scanalyzer, nitrogen deficiency, phenomics, *Sorghum*.

## Abstract

The use of high-throughput phenotyping systems and non-destructive imaging is widely regarded as a key technology allowing scientists and breeders to develop crops with the ability to perform well under diverse environmental conditions. However, many of these phenotyping studies have been optimized using the model plant *Arabidopsis thaliana*. In this study, The Plant Accelerator^®^ at The University of Adelaide, Australia, was used to investigate the growth and phenotypic response of the important cereal crop, *Sorghum bicolor* L. Moench and related hybrids to water-limited conditions and different levels of fertilizer. Imaging in different spectral ranges was used to monitor plant composition, chlorophyll, and moisture content. Phenotypic image analysis accurately measured plant biomass. The data set obtained enabled the responses of the different sorghum varieties to the experimental treatments to be differentiated and modelled. Plant architectural instead of architecture elements were determined using imaging and found to correlate with an improved tolerance to stress, for example diurnal leaf curling and leaf area index. Analysis of colour images revealed that leaf ‘greenness’ correlated with foliar nitrogen and chlorophyll, while near infrared reflectance (NIR) analysis was a good predictor of water content and leaf thickness, and correlated with plant moisture content. It is shown that imaging sorghum using a high-throughput system can accurately identify and differentiate between growth and specific phenotypic traits. R scripts for robust, parsimonious models are provided to allow other users of phenomic imaging systems to extract useful data readily, and thus relieve a bottleneck in phenotypic screening of multiple genotypes of key crop plants.

## Highlight

A high-throughput imaging system was used to compare growth and architectural traits of sorghum grown under different environmental conditions, and validated against traditional measures of plant performance and composition.

## Introduction

In order to feed an increasing global population, crops must be bred that are able to withstand and perform well under adverse environmental conditions, are resistant to herbivores and pathogens, and have increased nutritional value, and a reduced demand for agricultural inputs such as a fertilizer (Tester and Langridge, 2010; Gregory and George, 2011). Gene sequencing of model and crop plants is proceeding at a rapid pace, but the translation of such data into the identification of desirable traits is constrained by the lack of knowledge of the associated phenotypes. High-throughput phenotypic analysis systems have the potential to alleviate this major bottleneck, but relatively few studies of this type have been carried out using crop plants (Cabrera-Bosquet *et al.*, 2012; Walter *et al.*, 2012; Yang *et al.*, 2013).

Sophisticated imaging technologies and platforms that measure plant growth and performance in an automated, high-throughput, non-destructive manner use cameras operating in the visible light range that generate two-dimensional images to track plant growth and health (Rajendran *et al.*, 2009; Arvidsson *et al.*, 2011; Golzarian *et al.*, 2011). Phenotypic databases are rapidly growing for the genetic model plant *Arabidopsis* (Arvidsson *et al.*, 2011; Pieruschka and Poorter, 2012), but there is an unmet need for similar databases to be developed for major grain crops, such as wheat, barley, and sorghum. For high-throughout phenotyping to become routinely adopted, screening protocols and data analysis pipelines need to be established, including robust and parsimonious models that can be applied with confidence to new experimental data. It is important that the outputs from these imaging platforms are bench-marked against traditional measures, so that they can be integrated with the wealth of phenotypic data already accumulated over many years of crop breeding. This requires that the models be experimentally verified for particular crops and a range of environmental conditions (Furbank and Tester, 2011; Fiorani *et al.*, 2012).

Measurement of plant growth has traditionally been labour intensive, for example destructively harvesting plants at specific time points to provide intermittent quantification of certain parameters such as total biomass or leaf area. Such data may be imprecise because they rely on means of subsets of plants at each harvest rather than following growth trajectories of individuals (Berger *et al.*, 2012). Imaging makes possible the measurement of dynamic changes in plant form, based on parameters such as leaf area index (LAI; leaf area per square metre), tillering, and compactness, on large numbers of individuals over time, raising the possibility of identifying ‘hidden traits’. Leaf rolling, an important trait for assessing drought tolerance in cereals (Richards *et al.*, 2010), is traditionally estimated by using a visual score or by assessing a single leaf (O’Toole and Cruz, 1980), but it is difficult to quantify numerically (Premachandra *et al.*, 1993). Imaging plants at different times of day or over prolonged periods of water stress could allow such measures to be quantified. Spectral imaging using fluorescence, near infrared reflectance (NIR), and thermal wavelengths can provide additional information about the health and composition of the plant (Romano *et al.*, 2011; Winterhalter *et al.*, 2011; Harbinson *et al.*, 2012), for example for quantifying the degree of chlorosis, necrosis, or senescence in response to biotic or abiotic stress (e.g. Rajendran *et al.*, 2009).

The advances of image-based phenotyping are only now being applied to grain crops in complex genotype × phenotype ×environment interactions. While experimental protocols and image and data analysis are beginning to be validated for small grain crops, such as barley and rice (e.g. Richards, 1991; Rajendran *et al.*, 2009; Golzarian *et al.*, 2011; Hairmansis *et al.*, 2014), there are clear differences in plant architecture from that seen in large C_4_ crops such as maize and sorghum that may be more suited to the novel traits made possible by the imaging software. In this study, the focus is on establishing experimental protocols for non-destructive phenotyping of sorghum, which to the authors’ knowledge has not previously been studied using image-based phenotyping. Morphological measurements and spectral imaging not used previously with small cereals are introduced. Grain sorghum (*Sorghum bicolor* L. Moench) and related forage hybrids were grown under different levels of nitrogen and water supply, and growth rates and composition measured by conventional destructive harvests were compared with data gathered from a system designed for high-throughput imaging. Sorghum has been identified as a crop of the future, owing to its ability to tolerate drought and high temperatures, the rich genomic resources available for cultivated and wild relatives (Paterson *et al.*, 2009; Mace *et al.*, 2013), and its potential as a bioenergy crop (Rooney *et al.*, 2007; Wang and Brummer, 2012). Sorghum is also a particularly useful crop for studying the interaction between morphology and nitrogen use efficiency, as a significant proportion of leaf nitrogen is present as nitrate and the cyanogenic glucoside, dhurrin (Gleadow and Møller, 2014).

## Materials and methods

### Plant material and experimental design

The experimental set-up was divided into two parts (see Supplementary Table S1 available at *JXB* online). The first experiment was a nitrogen trial. Two different hybrids (HyA and HyB) of forage sorghum [*Sorghum bicolor* (L.) Moench×*Sorghum bicolor* (L.) Moench ssp. *drummondii* (Steud.) de Wet. (syn. *Sorghum sudanense* (Piper) Stapf.] were grown under different levels of nitrogen. In the second experiment, a single hybrid (HyA) was compared with a variety of grain sorghum [*Sorghum bicolor* (L.) Moench (Sb)] grown under well-watered and water-limited conditions. Both experiments were conducted at The Plant Accelerator^®^ Adelaide, Australia (34°58’17.00’’S, 138°38’23.00’’E) during summer in successive years (nitrogen trial, March 2011; watering trial, January/February 2012) with natural daylight and a photoperiod of 12h and 14.5h, respectively. Average light intensity over a sunny day during this period is ~700 μmol m^–2^ s^–1^ at plant level. Temperature in the greenhouse was 28 °C day/18 °C night on a sinusoidal cycle and measured every 5min with a Hobo^®^ data logger (Onetemp, Marleston, Australia). Seeds were germinated in 2.5 litre pots (150mm diameter×200mm deep). At the three-leaf stage (~2 weeks old), seedlings were thinned to one per pot and pots transferred to the conveyor system for automated imaging. At the end of the experimental period, samples of the plants were destructively harvested for biomass, morphometric, and chemical analyses.

### Experiment 1: nitrogen experiment

Plants were grown at three different levels of nitrogen. The low and moderate nitrogen growth conditions were achieved by using a nutrient-free coco-peat potting mix and watering three times per week with modified half-strength Hoagland’s solution containing either 1.5mM N (LowN) or 4.5mM N (MidN) (*n*=6). The high nitrogen (HighN) treatment was achieved by growing plants (*n*=3) in coco-peat potting mix containing ~3g l^–1^ slow release fertilizer [Osmocote^®^mini 3–4 month (16-3-9+trace elements)] equivalent to half-strength Hoagland’s nutrient solution containing ~20mM N, based on the amount of nitrogen in the fertilizer added to the pots and confirmed by the level of foliar N achieved (see Supplementary Table S5 at *JXB* online; R. Gleadow, unpublished data). After 4 weeks, all plants from each treatment were harvested. Height was measured as the distance between the base of the plant at the soil surface and the ligule of the first fully expanded leaf, where the blade subtends the stem. Leaves were counted, detached from the stem distal to the ligule, and total leaf area was measured using a LI-COR 3000 leaf area meter (LI-COR Lincoln, NE, USA). The shoot was removed at soil level, taking care to exclude any adventitious roots, and divided into leaves and stems. Anatomically, the stem comprised both the stem and the leaf sheaths of the leaves (O’Donnell *et al.*, 2013). Plant tissues were dried at 50 °C for 14 d, finely ground in a Mixer Mill 301 (Retsch, Dusseldorf, Germany), and used for further chemical analyses (see below).

### Experiment 2: watering experiment

Plants were grown in a 50:50 (v/v) coco-peat mix:UC Davis potting mix with added slow-release fertilizer (as above) and maintained at a gravimetric water content of ~20% (w/w). After 2 weeks, 10 plants of each variety were destructively harvested for biometric analysis (*n*=10). The remaining plants were transferred to the automated conveyor system of The Plant Accelerator^®^ for imaging and growth analysis. Pots were weighed daily and water added to maintain a gravimetric water content of 15% (w/w) or 5% (w/w) in the well-watered (HighW; *n*=14) and water-limiting (LowW; *n*=14) treatments, respectively. Pots (*n*=4) with no plants were included in each treatment as controls to monitor water loss through soil surface evaporation. Pots were weighed before and after each watering to calculate cumulative water use. After 4 weeks, the plants in The Plant Accelerator^®^ were harvested as in experiment 1. Water use efficiency (WUE) was defined as the above-ground biomass produced per mass of water consumed (i.e. evaporation and transpiration) during the corresponding growth period (Richards, 1991).

### Growth analysis of destructively harvested material

The following growth parameters were calculated from the destructive harvests: leaf mass per area (LMA; ratio of dry leaf mass to leaf area) and relative growth rate (RGR, g d^–1^), hereafter RGR(biomass). RGR(biomass) was calculated according to Hunt (1982):

$$\text{R}=\left({\text{ln W}}_{\text{2}}–{\text{ln W}}_{\text{1}}\right)/\left(t_{\text{1}}–t_{\text{2}}\right)$$ (1)

where W_1_ and W_2_ are the initial and final biomass at the beginning (*t*_1_) and end (*t*_2_) of the measurement period, respectively. In the first experiment, W_1_ was taken as the average seed weight of each hybrid (HyA and HyB, *n*=50). In the second experiment, W_1_ was the average dry weight of a set of plants (*n*=5) selected randomly prior to the start of the watering treatments. Moisture content (%) and dry matter (%) were derived from the difference in mass between fresh and dried tissue.

### Chemical analysis

All analyses were performed using finely ground leaf tissue. Total elemental nitrogen concentration was measured on 5mg samples using an Elementar Vario Micro Cube, CNS analyser with acetanilide (Merck, Australia) as the internal standard. The total amount of hydrogen cyanide evolved from the hydrolysis of dhurrin (the cyanide potential, HCNp), was determined using 10mg of leaf samples following Gleadow *et al.* (2012). Total nitrate concentration was measured on 15mg samples using 96-well microtitre plates (O’Donnell *et al.*, 2013). Chlorophyll was measured following Burns *et al.* (2002).

### Image acquisition and image analysis

Plants were imaged three times each week (nitrogen experiment) or daily (watering experiment) using a Scanalyzer 3D imaging system (LemnaTec GmbH, Aachen, Germany) (Berger *et al.*, 2010). Five mega pixel colour images were recorded from the top of the plant (top view) and from the side of the plant at two different rotations (0 °, side view 1; 90°, side view 2). The recorded images were processed using LemnaGrid software (LemnaTec GmbH). In brief, a region of interest was defined to comprise the entire plant but cut out the visible parts of the imaging hardware (e.g. lifter/turner). Plants were separated from the imaging background using a nearest-neighbour colour classification. Noise was removed through erosion and dilatation steps before composing all parts identified as plant to one object. The size, height, and dimensions of the object were calculated and all three images were used to estimate the overall biomass of the plant and were compared with actual plant size determined on the destructively harvested plants. Conversion of pixel area to millimetres for the side view images was achieved by multiplying pixel number by the constant 0.273. Conversion of pixels from the top view camera accounted for the changing distance effect as the plant grew closer to the camera. The top view-specific constant was calculated by the following:

$$K_{\text{TV}}=\text{9}.\text{937E1}0^{-\text{5}}Y_{\text{ax}}+0.0\text{61936}$$ (2)

where *K*_TV_ is the top view-specific constant, converting pixel area to millimetres, and *Y*_ax_ is the mean centre of mass along the *y*-axis for the two side view images. Final calculation of leaf area was achieved by summing all the calibrated side view and top view images.

Plant size measured using imaging was compared with actual plant size determined on destructively harvested plants using linear regression (see ‘Statistical analysis’). In experiment 1, final leaf area (imaged) was compared with total leaf area (measured) and final above-ground biomass (measured). Height to the ligule of the first fully expanded leaf (i.e. at the point where the leaf blade meets the stem), a commonly determined agronomic trait, was compared with imaged maximum plant height (i.e. to the top of the plant) and height to the first ligule.

**Fig. 1.**
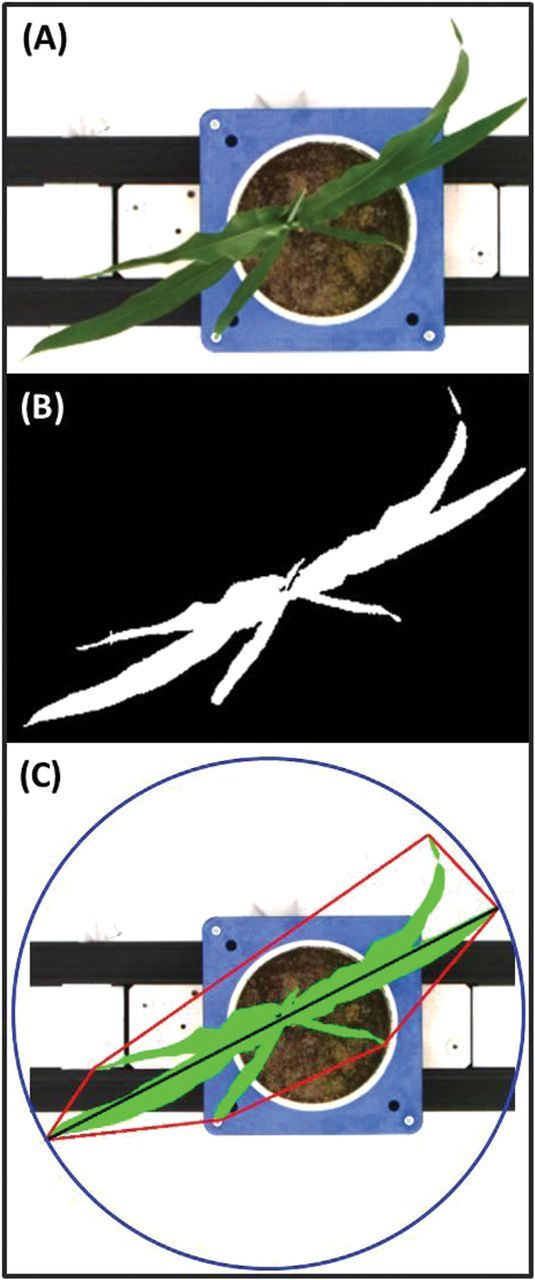
Example of image processing methods and derivation of geometric parameters. First, the foreground and background are separated in the raw image, as taken from above (A), using a nearest-neighbour colour classification, resulting in a binary image (B). Thereafter, the object (highlighted green; C) undergoes geometric measurements, such as calliper length (black line), convex hull (red line), and minimum enclosing circle (blue line). Compactness and surface coverage are measures of leaf coverage and are the ratio of object area (green) and convex hull or minimum enclosing circle area, respectively.

To estimate the degree of leaf rolling in the drought experiment, plants were imaged in the late afternoon (rolled leaves) and at pre-dawn the following day (unfolded leaves), 45 d after planting. Additional types of automated imaging analysis included the geometric parameters of convex hull (the smallest possible mathematically solved perimeter that envelopes the imaged plant), compactness (the ratio of leaf area per convex hull area), calliper length (the longest dimension of the canopy when viewed from above), circumference (the minimum circle that can enclose the plant), and surface coverage (the ratio of leaf area to the area of the minimum enclosing circle calculated from the top view image) (Fig. 1). Compactness and convex hull measure the degree of leaf area coverage, analogous to the agronomic measure of LAI. The degree of radial symmetry (eccentricity) was also determined.

### Colour and NIR imaging

Plants were imaged using light in the visible spectrum in RGB (400–700nm) and the NIR (750–1400nm). Greenness of the leaves was estimated by converting the images from the RGB to the Hue Saturation Intensity (HSI) colour management system using known algorithms with hue angle as an indication of leaf colour (Tackenberg, 2007). Leaf senescence was determined by categorizing leaf colour into healthy (green) and senescent (yellow) using the relative proportion of pixels in the RGB images (Rajendran *et al.*, 2009). NIR images from two side views (0 º, side view 1; 90º, side view 2) were acquired with the Scanalyzer 3D imaging system using an NIR-300 camera (Allied Vision Technologies GmbH, Stadtroda, Germany). The camera has a spectral sensitivity of 900–1700nm and an optical resolution of 320×256 pixels. The water absorption band at 1450nm is the strongest absorption feature in this spectral region, and the NIR signal has been used to estimate the water content in shoots (Seelig *et al.*, 2008). Since object recognition in the grey-scale NIR images is difficult, the identified object from the RGB images was used to create a mask for overlay with the NIR images. The mean grey value (8-bit scale) of the identified objects from both NIR images was calculated, where high values represent high reflectance and indicate low water content, while low grey-scale values represent high absorption and high water content.

### Selection of models

In order to assess plant growth through time, eight different published growth models were applied following visual assessment of the data (see Supplementary Fig. S1 at *JXB* online for workflow). Equations for all models tested are given in Supplementary Table S2. The suitability of each model was judged on how well it approximated the data based on best-fit analysis using two statistical methods, *R* ^2^ and Akaike information criterion (AIC) (see ‘Statistical analysis’, below). Confidence intervals (95%) were calculated using population prediction intervals (Paine *et al.*, 2012). Models requiring the least number of parameters for a similar degree of convergence were chosen (parsimony). The robustness of each model (i.e. the degree to which the model could be applied to multiple data sets and remain statistically valid) was tested.

Growth models were applied to height and leaf area data collected from the imaging and used to calculate relative growth rate, hereafter RGR(imaged). RGR(imaged) was then compared with RGR(biomass) and an area-based RGR [RGR(leaf area)] calculated using the average final and initial imaged leaf area, simulating the way that the RGR(biomass) is calculated from initial and final destructive harvests. Models were also applied to the increase in leaf area of individual plants over time, rather than to groups of plants, allowing the derivation of growth rates for individuals, known hereafter as RGR(individual). Absolute growth rate, AGR(imaged), was derived from RGR(imaged) by integrating the plant size equation with respect to time.

### Model algorithms

Non-linear regressions were implemented in R version 2.15.1 (R Development Team, 2011) using the gnls function in the nlme package. Self-starting functions were used where available in the base R packages (SSlogis, SSgompertz, SSfpl, SSRichards, SSweibull). Self-starting functions were created for the power law and beta function (see Supplementary Table S4 at *JXB* online). In experiment 1, since leaf area increase followed an exponential pattern, two non-asymptotic (exponential and power law) and one sigmoidal model were applied to the data. The exponential model was unable to converge on a solution for the HyB HighN group (Supplementary Table S3A). In contrast, the three-parameter logistic and power law models produced curves with similar *R* ^2^ and AIC values (Supplementary Table S3A) but the confidence intervals were smaller when a non-asymptotic power law model was applied (Equation 3). Therefore, for plants grown in the nitrogen experiment, the power law model was chosen for subsequent analyses such that:

$$M_{t}={\left(M_{0}^{1-\beta }+rt(1-\beta )\right)}^{1/1-\beta }$$ (3)

where *M*_*t*_ is the measured value at time *t*, *t* is the time after sowing, *M*_0_ is the measured value at time 0, *r* controls the growth rate, and β alters the progressive change in RGR.

In the water-limitation experiment, leaf area followed a sigmoidal pattern, such that leaf area plateaued towards the end of the sampling period. Six different sigmoidal growth models were therefore applied: Gompertz, Beta Function, Three- and Four Parameter Logistic (3PL and 4PL, respectively), Weibull, and Richards (Archontoulis and Miguez, 2013) (Supplementary Fig. S1 at *JXB* online). The 4PL, Beta, Weibull, and Richards models were unable to converge on a solution for all sorghum varieties and treatments (i.e. these models only gave solutions to most growth trajectories but not all; Supplementary Table S3B). In contrast, the 3PL and Gompertz models were able to converge on a solution for all varieties and treatments, following a similar trajectory with near identical values of *R* ^2^ and AIC values (Supplementary Table S3B). The Gompertz model differed from the 3PL model by having a higher maximum asymptote for shoot area and AGR towards the end of the experiment and, consequently, a higher standard error (Supplementary Table S3B; Asym value). Therefore, the 3PL model was chosen for subsequent growth analyses (Equation 4). Projected leaf area for the drought experiment was modelled using the asymptotic 3PL model function, following Paine *et al.* (2012) such that:

$$M_{t}=\frac{Asym}{1+e^{\frac{t_{mid}-t}{k}}}$$ (4)

where *M*_*t*_ is the measured value at time *t*, where *t* is the time after sowing, *Asym* is the carrying capacity (upper asymptote), and *k* controls the maximum growth rate.

### Statistical analysis

Specific models were selected based on confidence interval analysis (95%) calculated using population prediction intervals (Bolker, 2008; Paine *et al.*, 2012) in R (R Development Team, 2011). Equations for all models and a statistical comparison of them is given in Supplementary Table S2 at *JXB* online. The R scripts for the models used are given in Supplementary Table S3. Correlative analysis of the validation studies was made using linear regression in SigmaPlot 12^®^ (Systat Software Inc., San Jose, CA, USA). Biometric data and chemical composition of harvested plants were analysed using two-way (experiment 1) and one-way (experiment 2) analyses of variance (ANOVAs) with general linear models (GLMs) in SigmaPlot 12^®^. Data were tested for normality and log transformed if necessary to satisfy the assumptions of the statistical methods. Means that were significantly different were determined post-hoc, using Tukey’s pairwise comparisons; means annotated with the same letters in graphs and tables are not significantly different at the 95% level. Mean values in text and tables are followed by one standard error of the mean (±1 SE).

## Results

### Validation of phenotypic imaging measurement of growth indices

**Fig. 2.**
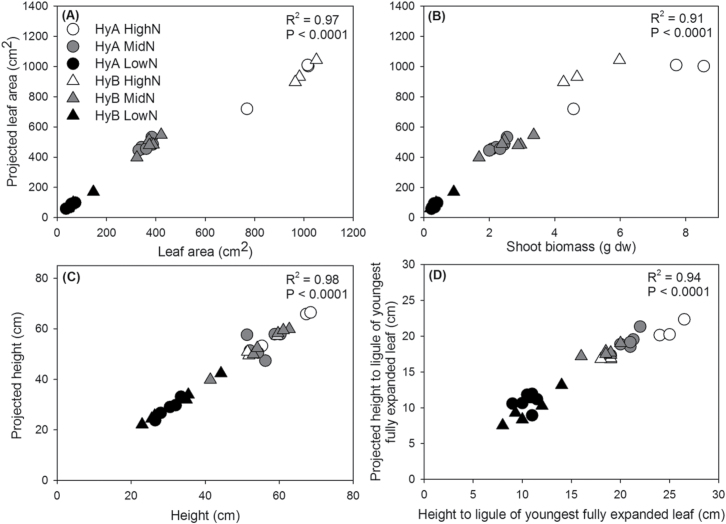
Relationship between measured leaf area (A), shoot biomass (B), plant height (C), and height to the ligule of the youngest fully expanded leaf (D) with projected measurements calculated from image analysis. Projected leaf area provides a strong indication of overall plant growth with a significant, positive correlation observed between projected and measured leaf area (*R* ^2^=0.97; *y*=0.94*x*+68.23) and above-ground biomass (*R* ^2^=0.91; *y*=138.69*x*+91.92). Projected height measurements positively correlated with plant height (*R* ^2^=0.98; *y*=0.98*x*–0.73) and height to ligule of the youngest fully expanded leaf (*R* ^2^=0.94; *y*=0.79*x*+2.24).

In order to assess whether the image acquisition system provided a true and accurate representation of the assessed features of sorghum, leaf area, plant height, and height to the ligule of the youngest fully expanded leaf recorded by traditional metrics were compared with the projected values acquired from image analysis. Two sorghum hybrid varieties, HyA and HyB, subjected to three different nitrogen treatments were examined. The relationship between projected and true leaf area was almost one to one (gradient=0.94; Fig. 2A; *R* ^2^=0.97; *P*<0.001), validating imaging as a highly accurate technique for measuring leaf area in sorghum. Strong positive correlations were also detected between projected leaf area and shoot biomass (Fig. 2B; *R* ^2^=0.91; *P*<0.001), projected and true plant height (Fig. 2C; *R* ^2^=0.98; *P*<0.001), and projected and true height measured to the ligule of the youngest fully expanded leaf (Fig. 2D; *R* ^2^=0.94; *P*<0.001). The latter is a useful and frequently measured agronomic trait. A strong positive correlation between projected leaf area and shoot biomass was also detected for Sb and HyA in the water-limiting experiment (Supplementary Fig. S2 at *JXB* online; *R* ^2^=0.97; *P*<0.001).

### Plant growth and biomass allocation in response to nitrogen and water deficiency

There was a strong treatment effect for all growth parameters derived from the destructive harvests in the first experiment in which two forage sorghum hybrids (HyA and HyB) were grown under high (HighN), medium (MidN), and low nitrogen (LowN) conditions (Table 1). For both hybrid varieties, lower values for all growth parameters were observed for plants grown at MidN and LowN (Table 1, Fig. 2A–C). The two hybrids displayed broadly similar growth patterns and biomass partitioning, with some differences at particular nitrogen levels (i.e. for some parameters the treatment×variety effect was significant). Dry matter content was 17% higher in HyB than in HyA plants grown at MidN (*P*<0.01), and there were some differences between the varieties in height and biomass allocation for some treatments, for example a greater proportion of biomass allocated to sheath mass in HyA in HighN treatment (Table 1). No significant differences between the two varieties in RGR(biomass) were detected.

**Table 1.** Growth parameters and descriptive statistics for the two varieties of sorghum hybrids (HyA and HyB) subjected to different nitrogen treatments

	HyA			HyB			Two-way ANOVA		
	High N	Mid N	Low N	High N	Mid N	Low N	V	T	V×T
Leaf area (cm^2^)	930±80	360±10	55±5	999±26	377±13	65±17	NS	***	NS
Height (cm)	64±4a	56±2b	29±1 c	54±3b	55±3b	32±3c	***	***	***
Shoot dry weight (g)	6.95±1.21a	2.27±0.09c	0.31±0.03 d	4.97±0.51b	2.62±0.24c	0.37±0.11d	NS	***	*
Leaf dry weight (g)	2.69±0.24	1.26±0.03	0.18±0.01	2.56±0.22	1.37±0.09	0.23±0.07	NS	***	NS
Sheath dry weight (g)	4.26±0.97a	1.01±0.05c,d	0.13±0.01 d	2.42±0.40b	1.25±0.16b,c	0.14±0.04d	NS	***	**
LMA (g^–1^ m^–2^)	28.80±0.52	35.08±0.37	33.67±1.30	25.54±1.85	36.07±1.30	34.60±1.18	NS	***	NS
Dry matter content (%)	19.11±2.19a,b,c	17.38±0.20b,c	20.04±0.59 a,b	14.89±1.13a	20.98±0.98a	20.5±0.46a,b	NS	**	**
RGR(biomass) (g g^–1^ d^–1^)	0.175±0.006	0.141±0.001	0.079±0.003	0.163±0.003	0.143±0.003	0.078±0.007	NS	***	NS

Mean ±standard error and significance of a two-way ANOVA are presented for each variety (V) and treatment (T).

*P<0.05; **P<0.01; ***P<0.001; NS, not significant.

LMA, leaf mass per unit area; RGR(biomass), relative growth rate calculated using results from the destructive harvest.

In the second experiment, Sb and one of the hybrids (HyA) were grown under well-watered (HighW) and water-limited (LowW) conditions. Overall, a strong treatment effect was observed in both varieties, with growth significantly reduced in both the Sb and HyA in the water-limiting treatment (Table 2). A significant variety×treatment interaction was observed, with leaf area and above-ground biomass significantly lower in Sb compared with HyA under both well-watered and water-limiting conditions (*P*<0.001; Table 2). Despite Sb having a smaller biomass than HyA, there was no significant difference in the traditional measure of RGR(biomass) (Table 2).

**Table 2.** Growth parameters and descriptive statistics for the two varieties of sorghum (*S. bicolor* or the HyA hybrid) grown under well-watered (HighW) and water-limited (LowW) conditions

	*S. bicolor*		HyA		Two-way ANOVA		
	HighW	LowW	HighW	LowW	V	T	V×T
Leaf area (cm^2^)	1870±40 b	634±36 d	3110±50 a	1260±70 c	***	***	***
Height (cm)	80±1	57±2	91±1	69±3	***	***	NS
Shoot dry weight (g)	9.5±0.3 b	3.8±0.2 d	14.8±0.4 a	5.8±0.3 c	***	***	***
Leaf dry weight (g)	5.9±0.2 b	2.3±0.1 d	8.3±0.2 a	3.6±0.2 c	***	***	**
Sheath dry weight (g)	3.6±0.2 b	1.5±0.1 c	6.5±0.3 a	2.2±0.1 c	***	***	***
LMA (g^–1^ m^–2^)	31.9±1.1	35.9±0.9	26.6±0.4	28.8±0.5	***	***	NS
RGR(biomass) (g g^–1^ d^–1^)	0.175±0.001	0.145±0.002	0.176±0.001	0.146±0.002	NS	***	NS
Dry matter content (%)	13.6±0.6	17.8±0.8	15.4±0.3	18.4±0.6	*	***	NS
Moisture content (%)	87.0±0.6	82.2±0.5	84.7±0.5	81.6±0.5	**	***	NS
WUE (g kg^–1^)	6.4±0.1	5.9±0.8	6.4±0.1	6.2±0.1	NS	**	NS

Mean ±standard error and significance of a two-way ANOVA are presented for each variety (V) and treatment (T).

*P<0.05; **P<0.01; ***P<0.001; NS, not significant.

LMA, leaf mass per unit area; RGR(biomass), relative growth rate calculated using results from the destructive harvest; WUE, water use efficiency.

WUE was estimated gravimetrically, measuring the amount of water lost from each pot over the course of the experiment and expressing it as a proportion of the final plant biomass. Average WUE was 6.4g kg^–1^ for both varieties when grown under well-watered conditions. Overall, there was a significant treatment effect (*P*=0.005), with a decreased WUE in the Sb variety to 5.9g kg^–1^ (±0.8) and a non-significant decrease to 6.2g kg^–1^ (±0.1) in HyA (*P*=0.264) (Table 2).

### Assessment of models and application to growth

**Fig. 3.**
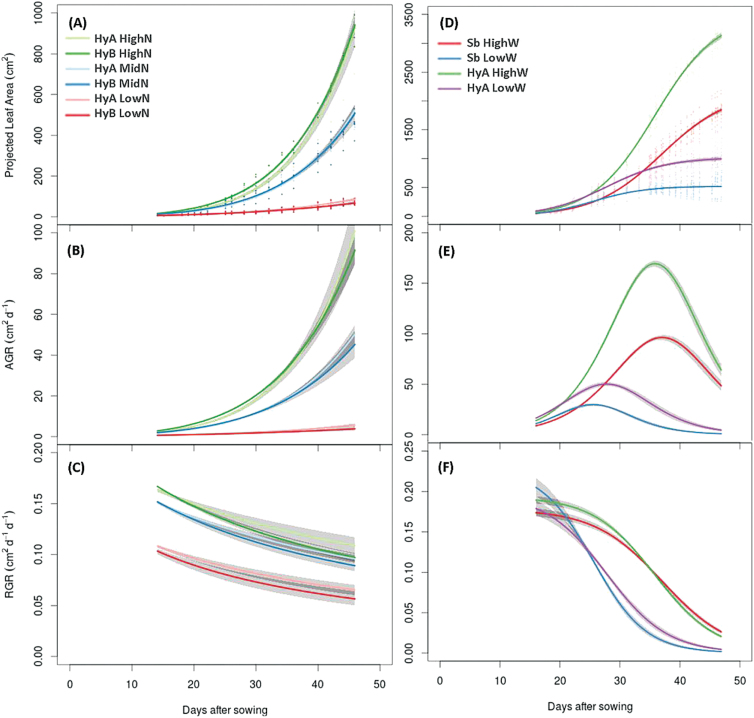
Calculated projected leaf area (A), absolute growth rate (B), and relative growth rate (C) for the non-asymptotic ‘nitrogen’ data set fitted with the power law model. Calculated projected leaf area (D), absolute growth rate (E), and relative growth rate (F) for the asymptotic ‘water-limited’ data set fitted with the three-parameter logistic model.

The models described by Equations 2 and 3 were used to calculate the RGR on a leaf area basis, RGR(imaged), for the nutrient and watering experiments, respectively. In experiment 1, the increase in leaf area followed an exponential pattern (Fig. 3A), and the non-asymptotic power law model was applied to the data (Equation 2). No significant differences between the two varieties in AGR(imaged) or RGR(imaged) were detected (Fig. 3B, C), consistent with RGR(biomass) calculated using harvested plants (Table 1).

In experiment 2, projected leaf area was modelled using the asymptotic 3PL model function (Equation 3). Under well-watered conditions, HyA initially had a higher RGR(imaged) than Sb, but decreased at a faster rate until day 35, when RGR(imaged) of HyA was significantly lower than that of Sb, and this lower growth rate was maintained until the end of the growing period (Fig. 3F). A similar trend was observed under water-limiting conditions, although the two plant varieties converged 10 d earlier, at day 25. Further variety differences were also observed in AGR(imaged), with HyA displaying a significantly higher AGR(imaged) compared with Sb under both well-watered and water-limited growing conditions (Fig. 3E). Due to the sigmoidal pattern of leaf area increase in this experiment, the AGR models exhibit a bell-shaped curve, with a clear point of inflection where the rate of leaf expansion (i.e. growth) reaches a maximum. Overall, both Sb and HyA reached maximum growth significantly earlier under water-limited conditions compared with the well-watered control treatment. Under well-watered conditions, there was no significant difference in the time at which growth peaked, with maximal growth at 35.9 d after sowing (±0.7 d) and at 37.0 d (±0.8 d) in HyA and Sb, respectively. In contrast, under water-limiting conditions, there was a significant difference between the two varieties, with growth peaking 2 d earlier in Sb than in HyA, at 25.6 d after sowing(±0.9 d) compared with the HyA variety at 27.8 d (±1.0 d).

To assess further the validity of the selected models for measuring growth, RGR(imaged) was compared with the dry mass of destructively harvested plants, RGR(biomass) (Supplementary Fig. S3 at *JXB* online). There was a strong correlation between RGR(imaged) and RGR(biomass) for plants grown under different nitrogen levels (*R* ^2^=0.93) (Supplementary Fig. S3A) but not for plants grown under water-limited conditions (*R* ^2^=0.83; Supplementary Fig. S3D). The initial mass used to calculate RGR(biomass) (W_1_, Equation 1) is necessarily the average biomass of a group of plants. In order to determine whether the difference between the two experiments was a limitation of the models or the result of relying on an initial pooled sample of plants, RGR(imaged) was also compared with RGR(leaf area), which uses the combined average initial leaf area but the final leaf area of individual plants. Again there was a good correlation between RGR(imaged) and RGR(leaf area) for the plants in the nitrogen experiment but not for those in the watering experiment (Supplementary Fig. S3B, E). However, when the initial leaf area for each individual plant was used to calculate growth, RGR(individual), instead of using a sample based on combined plants [i.e. RGR(leaf area)], a very high correlation was detected in both experiments (*R* ^2^=0.93–0.94). By following individual plants, it was possible to resolve differences in growth rate within the treatment groups (Supplementary Fig. S3C, F), as well as between treatments.

### Use of imaging to assess changes in plant architecture in response to water deficit

**Fig. 4.**
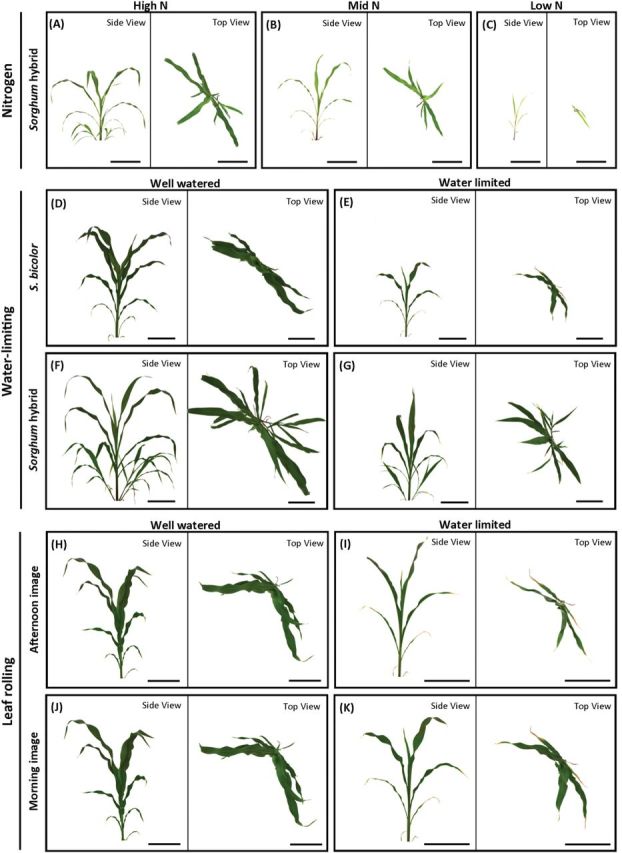
Images acquired of representative sorghum plants showing the effects of nitrogen deficiency (A–C) and water-limiting conditions (D–G) on plant architecture. Water limitation initiates leaf curling in sorghum plants, demonstrated by a 28% reduction in leaf area in drought-stressed plants (I, K) compared with a 4% increase in the control treatment (H, J). Scale bar=20cm.

Detailed analysis of images captured during the water-limiting experiment enabled accurate measures of plant architecture to be recorded, such as the type of symmetry, the area that the canopy occupies within a defined space (convex hull and compactness), and the size of the canopy (calliper length) (see Fig. 1), as well as traditional features such as height and tiller number. This type of detailed information is difficult and time-consuming to obtain manually. All plants in each experiment were analysed at the same developmental stage (eight-leaf stage). HyA had a significantly longer calliper length and a larger convex hull area than Sb, consistent with the larger leaf area (*P*<0.001; Table 3). In the water-limited experiment, only two Sb plants produced tillers, whereas all HyA individuals produced tillers (two under water-limited conditions and three under the well-watered conditions) (Fig. 4; Table 3). Despite the difference in tiller number, the degree of eccentricity (used as a measure of radial symmetry) was not significantly different between the Sb and HyA plants grown under well-watered conditions, but both varieties showed increased eccentricity in architectural form when water limited (*P*<0.05; Table 3).

**Table 3.** Geometric parameters extracted from image analysis of two varieties of sorghum (S. bicolor or the forage sorghum hybrid HyA) grown under well-watered (HighW) and/or water-limited (LowW) conditions

	*S. bicolor*		HyA		Two-way ANOVA		
	HighW	LowW	HighW	LowW	V	T	V×T
NIR	110.5±0.7 a	125.9±0.7 d	112±0.7 b	122.5±0.7 c	NS	***	***
Senescence (%)	2.5±0.1 a	6.0±1.1 c	3.6±0.2 b	3.5±0.3 b	NS	**	***
Mean HUE angle	87.0±0.6 b	84.2±0.7 a	85.0±0.7 a	86.3±0.7 a	NS	NS	**
Compactness	0.31±0.02	0.27±0.03	0.27±0.01	0.23±0.01	*	NS	NS
Convex hull area (cm^2^)	220±50 c	120±50 a	320±70 d	190±50 b	***	***	***
Eccentricity	0.49±0.05	0.62±0.06	0.40±0.06	0.55±0.05	NS	*	NS
Caliper length	85.7±2.5 c	52.3±4.6 a	110.6±2.1 d	72.1±1.7 b	***	***	**
Surface coverage	0.12±0.01	0.08±0.01	0.13±0.01	0.09±0.01	NS	***	NS
Tiller number	0.2±0.2 a	0.1±0.1 a	2.6±0.2 c	1.9±0.1 b	***	*	*

Mean ±standard error and significance of a two-way ANOVA are presented for each variety (V) and treatment (T).

*P<0.05; **P<0.01; ***P<0.001; NS, not significant.

NIR, near infrared reflectance; WUE, water use efficiency; WL, water-limited; WW, watered control.

**Fig. 5.**
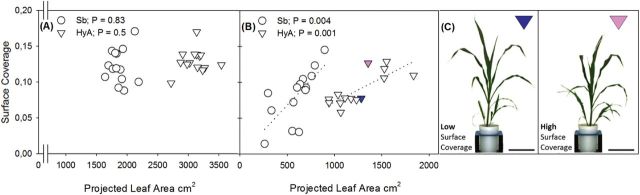
Relationship between plant performance as represented by the projected leaf area and surface coverage in sorghum plants grown under well-watered (A) and water-limiting conditions (B). Under well-watered conditions, no correlation was observed in the *S. bicolor* (Sb; circle) or hybrid (HyA; triangle) varieties (*P*=0.83 and 0.5, respectively). A positive correlation was observed under water-limiting conditions for the Sb (*P*=0.004; *R* ^2^=0.51; *y*=1.41e^–4^ *x*–2.38e^–3^) and HyA varieties (*P*=0.001; *R* ^2^=0.61; *y*=6.76e^–5^ *x*+4.65e^–3^). Anatomical differences within the plant line that cannot be distinguished by visual inspection alone, demonstrated by two representative HyA individuals of similar leaf area (C), with one individual possessing a lower (blue) or higher (pink) surface coverage. Scale bar = 20 cm

The descriptors ‘compactness’ and ‘surface coverage’ are derived from the ratio between top view leaf pixels and the convex hull, and the minimum enclosing circle, respectively and can be used as a proxy for LAI (see Fig. 1). While compactness appeared to be lower in Sb and HyA grown under water-limited conditions, the difference was not significant (Table 3). Surface coverage was significantly lower when plants were subject to water-limiting conditions (*P*>0.001; Table 3). Consistent with this was the highly significant correlation between surface coverage and final leaf area (i.e. plant size) in both varieties where water was limiting (Fig. 5B; *P*<0.001), but not under well-watered conditions (Fig. 5A). This newly derived leaf surface area–plant size parameter, here called ‘surface coverage’, appears to allow differences between individuals to be distinguished, with some individuals able to grow larger when subjected to water limitation than others (Fig. 5B). In the case of HyA, the surface coverage parameter clustered the water-limited individuals into two distinct groups. Such differentiation between groups could not be made by visual inspection alone (Fig. 5C).

### Leaf rolling and senescence in response to drought stress

The effect of leaf rolling on the diurnal variation in leaf area was quantified by imaging plants in the late afternoon (at ~17:00h) and early the following morning (at ~05:00h). Overall, there was a significant increase in the pre-dawn leaf area in both well-watered and water-limited plants. In well-watered plants, the increase was small, with leaf area increasing by ~3% and 6% in Sb and HyA, respectively. In water-limited plants, pre-dawn leaf area was ~28% greater compared with the late afternoon (Table 4; Fig. 4I,K).

**Table 4.** Comparison of the diurnal effect on mean leaf area (LA) ±s.e in two varieties of sorghum [*S. bicolor* (Sb) and the HyA hybrid] grown under either well-watered (HighW) or water-limited (LowW) conditions Images were captured in the late afternoon and the following morning.

Line	Treatment	17:00 h	05:00 h	Paired *t*-test	
		LA cm^2^	LA cm^2^	*P*	% LA increase
Sb	HighW	1760±40	1820±40	***	3.1±0.4
HyA	HighW	2890±50	3060±57	***	5.6±0.9
Sb	LowW	860±50	1210±70	***	28.7±1.1
HyA	LowW	470±20	660±30	***	28.2±1.1

Significance of a paired *t*-test and two-way ANOVA are presented. ***P<0.001.

When well watered, HyA had a significantly higher proportion of senesced leaf area compared with Sb, with a mean leaf senesced area of 3.6% and 2.5%, respectively (*P*<0.001) (Table 3). However, when grown under water-limiting conditions, the proportion of leaf senescence increased to 6% in Sb (*P*<0.001), whereas HyA maintained a similar proportion of senesced leaf area to the well-watered controls (3.5%; *P*=0.458). A two-way ANOVA of leaf senescence confirmed that the variety×treatment interactions were significant. The same parameters were used to examine differences between individual plants. Within Sb, leaf senescence was more pronounced in three individuals, with >10% of total leaf area considered senesced (average of 12.5%). Interestingly, unlike the other plants, the leaves of these three individuals also remained rolled when imaged in the early morning. Overall, the combination of the persistently rolled leaves and pronounced senescence in these apparently water-stressed individuals resulted in a reduction of the maximum leaf area by ~35% (Supplementary Fig. S4 at *JXB* online). Moreover, these phenotypic traits corresponded to yield, with these individuals also having the lowest biomass.

### Use of NIR image analysis to determine water status and leaf thickness

**Fig. 6.**
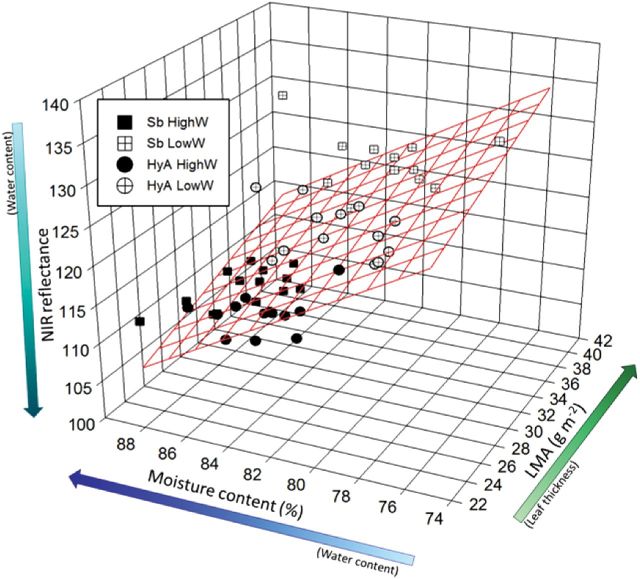
Examination of the variation in near infrared (NIR) reflectance with moisture content and leaf mass per unit area (LMA). A multiple regression analysis was performed (grid lines) using the combined data for *S. bicolor* (Sb, squares) and hybrid (HyA, circles) and the control (black) and drought-stressed (cross) treatments. The equation for the multiple regression is: NIR=228.3+(0.55×LMA)–(1.52×% H_2_O); (*R* ^2^=0.55; *P*<0.001).

NIR reflectance was significantly lower in well-watered plants, indicating a higher moisture density compared with water-limited plants (Table 3). This is consistent with the moisture content measured conventionally using the difference between the fresh and dry mass (*P*=0.016; Table 2), which, as expected, was higher in well-watered compared with water-limited plants (*P*<0.001; Table 2). Statistically significant variety and treatment interactions were also observed (Table 3; *P*<0.001). In well-watered plants, NIR reflectance was lower in Sb compared with HyA, whereas in plants from the water-limited treatment, NIR reflectance was higher in Sb (*P*<0.001; Table 3). This result is notable in that it is not consistent with the classically measured moisture content, where no varietal differences were observed (*P*=0.502; Table 2), and indicates that other factors, such as leaf thickness, may have contributed to the difference in NIR reflectance. To test this hypothesis, a multiple regression analysis using leaf mass per unit area (LMA; indicative of leaf thickness), moisture content, and NIR was performed, and a significant relationship (*P*<0.001) was detected (Fig. 6). LMA was significantly higher in Sb than in HyA in both treatment groups. Sb also showed a greater increase in LMA in response to water limitation (i.e. the variety×treatment interaction was significant) with a 12% increase (32±1 to 36±1cm^2^ g^–1^; *P*<0.001) compared with 8% in HyA (26.6±0.4 to 28.8±0.5cm^2^ g^–1^; *P*=0.02) (Table 2). This supports the hypothesis that NIR values are driven by a combination of both moisture content and leaf thickness. Together, these analyses demonstrate that, in this experiment, the difference in NIR reflectance between the two varieties under water-limiting conditions can be attributed to the differential response in LMA.

### Use of imaging and traditional measurements to describe aspects of chemical composition

Total chlorophyll concentration was measured using wet chemistry. There were no significant differences between the two forage sorghum varieties (HyA and HyB) or between forage sorghum (HyA) and grain sorghum (Sb) (Supplementary Tables S5, S6 at *JXB* online). Chlorophyll concentration was significantly higher in plants grown at progressively higher nitrogen levels, with a mean (combined data for both hybrids) of 4.6, 6.8, and 7.5mg g^–1^, respectively (Supplementary Table S5). No effect of drought on chlorophyll levels was detected.

**Fig. 7.**
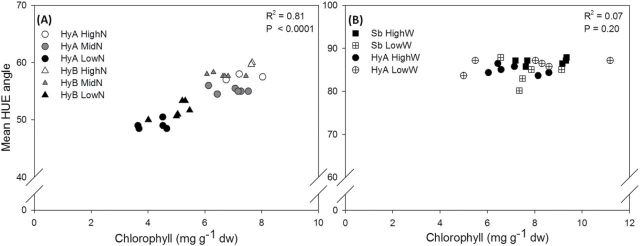
Relationship between mean hue angle and total chlorophyll concentration in sorghum. A significant correlation between mean hue angle and chlorophyll concentration within nitrogen treatments (A; *P*<0.0001; *R* ^2^=0.81; *y*=2.56*x*+37.58) but not for drought-induced stress (B; *P*=0.20; *R* ^2^=0.07; *y*=0.52*x*+81.25).

Chlorophyll concentration was compared with leaf greenness using the mean hue angle of individual images. In the nitrogen experiment, the treatment effect was highly significant for both mean hue angle (*P*<0.001) and chlorophyll concentration (*P*<0.001), with progressively lower measures of both chlorophyll and hue angle with decreasing levels of nitrogen (Fig. 7). Consistent with this, regression analysis revealed a highly significant positive correlation between chlorophyll concentration and mean hue angle (*P*<0.0001; Fig. 7A). In contrast, in the watering experiment, there were no significant effects of variety or treatment on chlorophyll concentration (*P*=0.255), and no correlation was detected between chlorophyll and mean hue angle (*P*=0.202; Fig. 7B). Image analysis, however, identified differences in hue angle between varieties and between treatment, and a highly significant variety×treatment interaction (*P*=0.008) (Table 3). Under well-watered conditions, the Sb variety possessed a significantly higher mean hue angle compared with HyA (*P*<0.001) but, when water limited, the hue angle only decreased significantly in Sb plants (*P*=0.013) but not in HyA (*P*=0.187).

Total nitrogen and the nitrogen-containing metabolites dhurrin and nitrate were measured on plants from all treatments, and several noteworthy findings were made. First, in the nitrogen experiment, foliar N was proportional to the availability of nitrogen. When data for the two varieties were combined, overall average foliar N concentrations of 1.2, 1.9, and 4.6% were found in leaves of plants grown at low, medium, and high nitrogen, respectively (*P*<0.001) (Supplementary Table S5 at *JXB* online). Secondly, HCNp was significantly lower in plants that were grown at LowN (0.13mg g^–1^) than those grown at MidN and HighN, which had overall mean concentrations of HCN of 0.43mg g^–1^ and 0.51mg g^–1^, respectively (Supplementary Table S5). Within nitrogen treatments, there were no significant differences in HCNp between the two varieties. Thirdly, foliar nitrate was significantly higher in HighN-grown plants, again with no significant differences between varieties. At HighN, foliar nitrate concentration was 11.7±0.5mg g^–1^ and 10.7±0.5mg g^–1^ for HyA and HyB, respectively, more than double that of plants grown at MidN (4.8±0.5 and 3.9±0.3mg g^–1^) and LowN (4.7±0.6 and 4.4±0.3mg g^–1^) (Supplementary Table S5). Fourthly, in the water-limiting experiment, HCNp, nitrate, and foliar N were all significantly higher in water-limited compared with well-watered plants (Supplementary Table S6). Mean HCNp was 0.86mg g^–1^ in water-limited Sb and HyA plants compared with 0.30±0.05mg g^–1^ in well-watered Sb plants and 0.51±0.05mg g^–1^ in well-watered HyA plants (*P*<0.001). Foliar nitrate concentration was also higher in water-limited plants, increasing from 2.5mg g^–1^ to 9.9mg g^–1^ in HyA, and more than doubling from 6.6mg g^–1^ to 16.2±1.5mg g^–1^ in Sb (Supplementary Table S6).

## Discussion

### Validation of image-based phenotyping to measure growth

Imaging-based phenotyping collects information that can be used to make inferences about plant growth, architecture, and composition on large numbers of plants over time. The power of high-throughput plant phenotyping is in the generation of large amounts of data. However, while the technology has a great potential for assisting in plant breeding (e.g. Walter *et al.*, 2012), its impact is limited by a lack of simple algorithms to extract and explore relevant information on specific plant phenotypes (Berger *et al.*, 2010). The aims of the experiments described here were: (i) to validate the use of continuous imaging as an alternative to destructive harvest and thus establish a protocol suitable for future high-throughput studies; (ii) to develop and apply robust, parsimonious models to compare the growth, composition, and shape of varieties of sorghum; and finally (iii) to compare the partitioning of nitrogen to nitrate and dhurrin in plants grown at different levels of nitrogen or water supply.

A good correspondence between projected and actual leaf area has been reported previously, but only for small or young plants in the exponential growth phase (Tackenberg, 2007; Fiorani and Schurr, 2013). Moreover such experiments have focused on model plants such as *Arabidopsis*, or relatively small cereal crops such as wheat and barley (Golzarian *et al.*, 2011). A strong positive correlation was found between projected leaf area and above-ground biomass, height, and true leaf area up to a size of >1000cm^2^ (Fig. 2). A study on the wild wheat, *Triticum monococcum* spp. *monococcum*, in contrast, found that leaf area prediction > 100cm^2^ became less accurate (Rajendran *et al.*, 2009). The higher accuracy in the present study compared with that of Rajendran *et al.* (2009) is probably due to species-specific differences in plant architecture. Nevertheless, the difference between these two species, both grasses, highlights the importance of calibrating and tailoring the image capture and analysis for particular species.

An advantage of image-based phenotyping is the capability for large numbers of plants to be screened over time in a dose–response curve manner (Poorter *et al.*, 2012). This is particularly important for studies where subtle or transient differences in growth occur. Traditional growth models, based on comparisons of destructively harvested plants at distinct time intervals (Evans, 1972), assume a constant relative rate of growth between the chosen time points, which may not be true. Plant responses to environmental changes tend to be non-linear and dynamic, and rates of biomass accumulation as a function of total biomass typically slow down in the course of plant ontogeny. The RGR output from the models used here showed significant correlation with the RGR measured by traditional destructive harvests, although the strength of the correlations varied. The two models applied to the data generated here (exponential and asymptotic) are applicable to many plant growth experiments, simplifying model selection for future studies (see Supplementary Fig. S1 at *JXB* online).

### Analysis of environmental effects on growth using image analysis and traditional metrics

Plants with access to ample fertilizer and water are usually taller, and have a higher RGR, greater total leaf area, and higher leaf mass fraction (Poorter *et al.*, 2012), as was observed here for sorghum. Growth and biomass partitioning traits measured by destructive harvesting revealed few differences (Table 1). However, imaging analysis and modelling showed that the HyA variety tended to have a higher RGR and AGR compared with HyB at all nutrient levels (Fig. 3). In the nitrogen fertilizer experiment, plants were harvested at the six-leaf stage (Vanderlip, 1993) and were still in the exponential growth phase. A longer experiment may have detected further differences between the two forage sorghum varieties in addition to the treatment effect.

In the water-limiting experiment, the growth period was extended until plants had reached the eight-leaf developmental stage (see Fig. 4A and F for size comparison between the two experiments), where leaf area accumulation transitioned from the exponential phase to the plateau phase (Fig. 3D) and the maximum AGR was reached (Fig. 3E). This point of inflection may reflect when a plant shifts from vegetative to reproductive growth. The timing of the shift can be regarded as a developmental trait (Shi *et al.*, 2013), but is difficult to pinpoint using traditional methods, particularly when plants are subjected to certain stress treatments (Shi *et al.*, 2013). Despite clear overall differences in plant size between the Sb and HyA varieties used in the water-limiting experiment, there was no difference in the point at which maximum growth was reached under well-watered conditions. Under water-limiting conditions, however, Sb reached maximum growth earlier than HyA (Fig. 3A). This shift in developmental response may be due to the small, but significant size differences in leaf area between the two varieties at the commencement of the watering treatment. Modelling revealed that HyA had a higher initial RGR than Sb under well-watered conditions, while RGR of Sb was higher when plants were water limited (Fig. 3). RGR(biomass) calculated using traditional endpoint harvesting methods could not resolve these differences.

Early seedling vigour and growth under well-watered conditions is often overlooked using classical selection based on manual analyses, despite its importance to later developmental stages and the inherent potential to improve crop yields (Richards and Lukacs, 2002). The ability for a seedling to generate leaves quickly is thought to be important in increasing resilience to drought stress later in development (Ludwig and Asseng, 2010; Richards *et al.*, 2010). Analysis of the images collected during the watering experiment detected treatment-affected differences in growth patterns within a few days of the start of the treatments in both experiments. The later time point at which the HyA line reached maximum growth in response to water limitation, for example, was consistent with enhanced seedling vigour compared with Sb. Imaging may thus make it possible to evaluate the response of plants to different application of nutrients at an early stage of development, reducing the duration of screening studies.

Phenotyping platforms that identify and differentiate between small differences in the responses to growth conditions may facilitate the selection of individual plants with traits of interest within large populations of plants that would otherwise be considered phenotypically identical if assessed using classical metrics. To assess whether a greater leaf area at the start of the water-limiting experiment affected the growth and response of individual sorghum plants at a later stage, the 3PL model was applied to the four largest and four smallest individual sorghum plants within the Sb and HyA lines, and the time point at which maximum absolute growth was reached (i.e. the point of inflection; Supplementary Fig. S3 at *JXB* online) was compared. Interestingly, no differences in the timing or magnitude of absolute growth rate between the smallest and largest individuals were observed when plants were water limited (Supplementary Fig. S5 at *JXB* online). However, when the three intermediately sized HyA and Sb seedlings were modelled (3PL), the point of inflection was both higher and later under water-limiting conditions (Supplementary Fig. S3), suggesting that there may be trade-offs between size and tolerance to low water.

### Evaluation of image-based phenotyping as a technique for assessing plant morphology under water-limiting conditions

Changes to plant morphology in response to water limitation result primarily in a smaller leaf area, through reduced tillering, slower leaf emergence, smaller leaves, altered leaf angle, leaf rolling, and ultimately senescence. In the watering experiment, Sb and HyA first responded to water limitation with a decrease in the rate of leaf appearance and growth, followed by a reduction in leaf area through changes in architecture and leaf rolling. The degree of leaf rolling is cumbersome to measure manually, necessitating regular measurements of leaf width, or the use of indirect ranking systems (O’Toole and Cruz, 1980). By imaging plants during the day, when they were more stressed, and also at night, when less stressed, it was possible to calculate the degree of leaf rolling and that it effectively reduced leaf area by 28% (Table 4). This does not necessarily mean that growth rates decreased to the same extent, because in sorghum the exposed abaxial surface is capable of high photosynthetic rates, minimizing any growth sacrifice (Lopes *et al.*, 2011). It was also shown that under the conditions imposed here, leaf rolling can be reversible on relatively short time scales. Importantly, those individuals that did not manage to unroll their leaves in the morning also had the lowest final biomass. Leaf rolling has been used to estimate leaf water potential and leaf diffusive resistance, for example in rice (O’Toole and Cruz, 1980; Blum *et al.*, 1989). The ability to automate the measurement of leaf rolling, and quantify it, may allow this to become a routine way to identify individuals responding poorly to water deficits.

Assessment of different growth parameters and characteristics available from the image analysis algorithms enabled further dissection of different traits, but to date there has been little discussion of the physiological relevance of these ‘digital’ traits. In classical studies, leaf area measurements are carried out by passing leaves through a leaf area meter, flattening each leaf in the process, and is thus not necessarily reflective of the area displayed for light interception. Novel image analyses of plant architecture such as convex hull and compactness provide indices of canopy cover and proxies for traditional measures such as LAI (leaf area per unit growth area). Here, compactness and convex hull were not found to correlate with plant growth under well-watered or water-limiting conditions. The parameter of ‘surface coverage’, however, could be used to identify individuals within both the Sb and HyA lines that accumulated a greater leaf area (Fig. 5) and biomass (data not shown) under water-limiting conditions. A high rate of canopy closure is related to crop WUE and yield, and is associated with an increased rate of tiller appearance rather than leaf expansion (Richards, 1991). The rate of appearance and number of tillers, in turn, vary with genotype and water availability. Image-based phenotyping enables individuals within inbred commercial lines that differ in otherwise unidentifiable traits to be distinguished, presenting new targets for crop improvement.

### Analysis of chemical composition grown under different environmental conditions

The nitrogen treatments used in the present study effected changes in foliar N to within the range expected from earlier studies (O’Donnell *et al.*, 2013; Miller *et al.*, 2014), corresponding to levels considered as deficient (1% N), marginal (2% N), and high (4% N) for plant growth (Reuter *et al.*, 1997). Nitrogen in excess of the requirements of primary metabolism appears to be first allocated to dhurrin and then to NO_3_. As previously reported, no significant difference in HCNp was detected between HyA and HyB in well-fertilized plants (Miller *et al.*, 2014). In sorghum, as in many other plants, there is a positive correlation between N availability, foliar N, and cyanogenic glucoside concentration (HCNp) (Gleadow and Møller, 2014). Consistent with this, foliar HCNp was significantly lower in plants grown in the LowN treatment. There was, however, no difference in foliar HCNp between MidN and HighN, even though leaf N doubled (Supplementary Table S5 at *JXB* online). It appears that additional nitrogen was allocated to nitrate, since foliar nitrate concentration was higher in plants from the HighN treatment, but not significantly different between the LowN and MidN treatments. Given that both foliar HCNp and nitrate were high in plants from the HighN treatment, it is possible that both may contribute to sorghum toxicity under highly fertilized growth conditions. Cyanogenic plants typically contain higher concentrations of cyanogenic glucosides when subject to drought or when experiencing osmotic stress (Neilson *et al.*, 2013; Gleadow and Møller, 2014). Here it was found that plants had higher foliar nitrate levels as well as higher HCNp when grown under water-limiting conditions, raising the possibility that toxicity of drought-stressed forage sorghum could be exacerbated by higher nitrate levels.

### Evaluation of usefulness of spectral analysis for estimating chemical composition

Spectral analysis has been used to provide information about chlorophyll content, rates of senescence (Kipp *et al.*, 2014), nitrogen deficiency (Rodriguez *et al.*, 2006), water content (Munns *et al.*, 2010), and WUE (Elsayed *et al.*, 2011), with varying degrees of accuracy being reported (Rodriguez *et al.*, 2006). Leaf yellowing is often the first visual cue to demonstrate that senescence has begun, and is correlated with chlorophyll degradation (Munne-Bosch and Alegre, 2004). The strong correlation between ‘greenness’ and chlorophyll concentration in plants growing at different levels of nitrogen validates the use of this trait. Although a similar correlation was not detected in plants subject to different watering regimes, the difference is probably due to the sampling method. Chlorophyll concentration was measured using leaf discs excised from the first fully expanded leaf, whereas ‘greenness’ was determined by analysing the colour composition of whole plants. While ‘greenness’ showed that the water-limited plants were clearly senescing, the first fully expanded leaf remained green and functional. Daily analysis of images of whole plants has the potential to pinpoint the onset of senescence removing risk of bias.

Analysis of reflectance at other wavelengths has proved useful for determining concentrations of other plant metabolites without the need for wet chemistry, with varying degrees of success (Heraud *et al.*, 2007; Munns *et al.*, 2010; Fox *et al.*, 2012). Measurements of reflectance at multiple wavelengths have been effectively used for measuring water content (Seelig *et al.*, 2008), but leaf thickness as well as water content will influence NIR reflectance (Fig. 6; Supplementary Fig. 6 at *JXB* online). Unlike in the study of Fox *et al.* (2012), it was not possible here to detect any correlation between NIR reflectance and dhurrin levels on dried tissue. In the present study, the dhurrin signal would probably be masked by the water signal.

### Conclusions

Image-based phenotyping is a powerful tool for analysing the nuances of plant growth and development. This study showed that minor differences in plant size and RGR can be resolved over time, thus increasing the power of phenotypic assessement over classical methods. In addition, transient phenotypes, such as leaf rolling, can be identified and quantified. By combining measurements of growth, architecture, and physiological aspects such as water content and WUE in one phenotyping protocol, the measurement of multiple, distinct phenotypic traits in parallel becomes possible. The study presented here, from phenotyping to data analysis, demonstrates the value of high-throughput phenotyping systems for screening sorghum, thus helping to identify individuals and varieties with traits contributing to stress tolerance or improved yield potential.

## Supplementary data

Supplementary data are available at *JXB* online.

Figure S1. Schematic overview for model selection and analysis.

Figure S2. Relationship between shoot biomass and projected leaf area calculated from image analysis for Sb and HyA plants subject to high and low water conditions.

Figure S3. Assessment of relative growth rate derived from the power law and 3PL models selected for the nutrient- and water-limiting experiments.

Figure S4. Phenotypic response in a representative *Sorghum bicolor* plant subjected to limited watering over four weeks as observed in images taken from above and from the side.

Figure S5. Effect of seedling size (small, intermediate, and large) on HyA and Sb growth under high and low watering regimes.

Figure S6. Differences in NIR reflectance, moisture content, and leaf mass per area in Sb and HyA plants subjected to well-watered and water-limiting conditions.

Table S1. Summary of the two experiments including cultivars and the dates of sowing and imaging.

Table S2. Summary of plant traits derived using LemnaGrid software using phenotypic imaging recorded in The Plant Accelerator^®^.

Table S3. Growth curve parameters for the modelled projected shoot area versus days after sowing for (A) the nitrogen and (B) the watering experiment with the calculated *R* ^2^ values. (C) Equations for eight models used to assess the growth of sorghum in two sets of data obtained from The Plant Accelerator^®^.

Table S4. R scripts used to calculate the fitted growth models, absolute growth rates, and relative growth rates along with the 95% confidence intervals for sorghum varieties used in the nitrogen and water-limiting experiments.

Table S5. Chlorophyll, HCN, and nitrate, concentration, and total nitrogen allocation in the leaf tissue of two hybrid sorghum varieties grown under different nitrogen conditions.

Table S6. Chlorophyll, HCN, and nitrate concentration, and total nitrogen allocation in the leaf tissue of well-watered and drought-stressed Sorghum bicolor and forage sorghum HyA.

Supplementary Data

## Abbreviations:

AGR: absolute growth rate
AIC: Akaike information criterion
RGR: relative growth rate
RGR(biomass): relative growth rate calculated from the destructive harvest
RGR(imaged): relative growth rate calculated from the image data
RGR(individual): relative growth rate calculated from the imaged data using the initial leaf area of individual plants
RGR(leaf area): calculated using the average final and initial imaged leaf area.
