# Supplementary material for: Utilization of a high-throughput shoot imaging system to examine the dynamic phenotypic responses of a C4 cereal crop plant to nitrogen and water deficiency over time
Source: J Exp Bot. 2015 Feb 19;66(7):1817–32. doi: 10.1093/jxb/eru526 (PMC4378625; doi:10.1093/jxb/eru526)
Supplement: Supplementary Data [file supp_66_7_1817__index.html]

Utilization of a high-throughput shoot imaging system to examine the dynamic phenotypic responses of a C4 cereal crop plant to nitrogen and water deficiency over time — Utilization of a high-throughput shoot imaging system to examine the dynamic phenotypic responses of a C4 cereal crop plant to nitrogen and water deficiency over time — Supplementary Data 

# Utilization of a high-throughput shoot imaging system to examine the dynamic phenotypic responses of a C4 cereal crop plant to nitrogen and water deficiency over time

## Supplementary Data

Data files

**Files in this Data Supplement:**

- Supplementary Data - Supplementary Data
