# Supplementary material for: Utilization of a high-throughput shoot imaging system to examine the dynamic phenotypic responses of a C4 cereal crop plant to nitrogen and water deficiency over time
Source: J Exp Bot. 2015 Feb 19;66(7):1817–32. doi: 10.1093/jxb/eru526 (PMC4378625; doi:10.1093/jxb/eru526)
Supplement: Supplementary Data [file supp_eru526_jexbot136184_file001.pdf]

Utilization of a high-throughput shoot imaging system to examine the dynamic phenotypic responses of a C4 cereal crop plant to nitrogen and water deficiency over time

E.H. Neilson, A.E. Edwards, C.K. Blomstedt, B. Berger, B. Lindberg Møller, R.M. Gleadow

SUPPLEMENTARY INFORMATION

SUPPLEMENTARY FIGURES

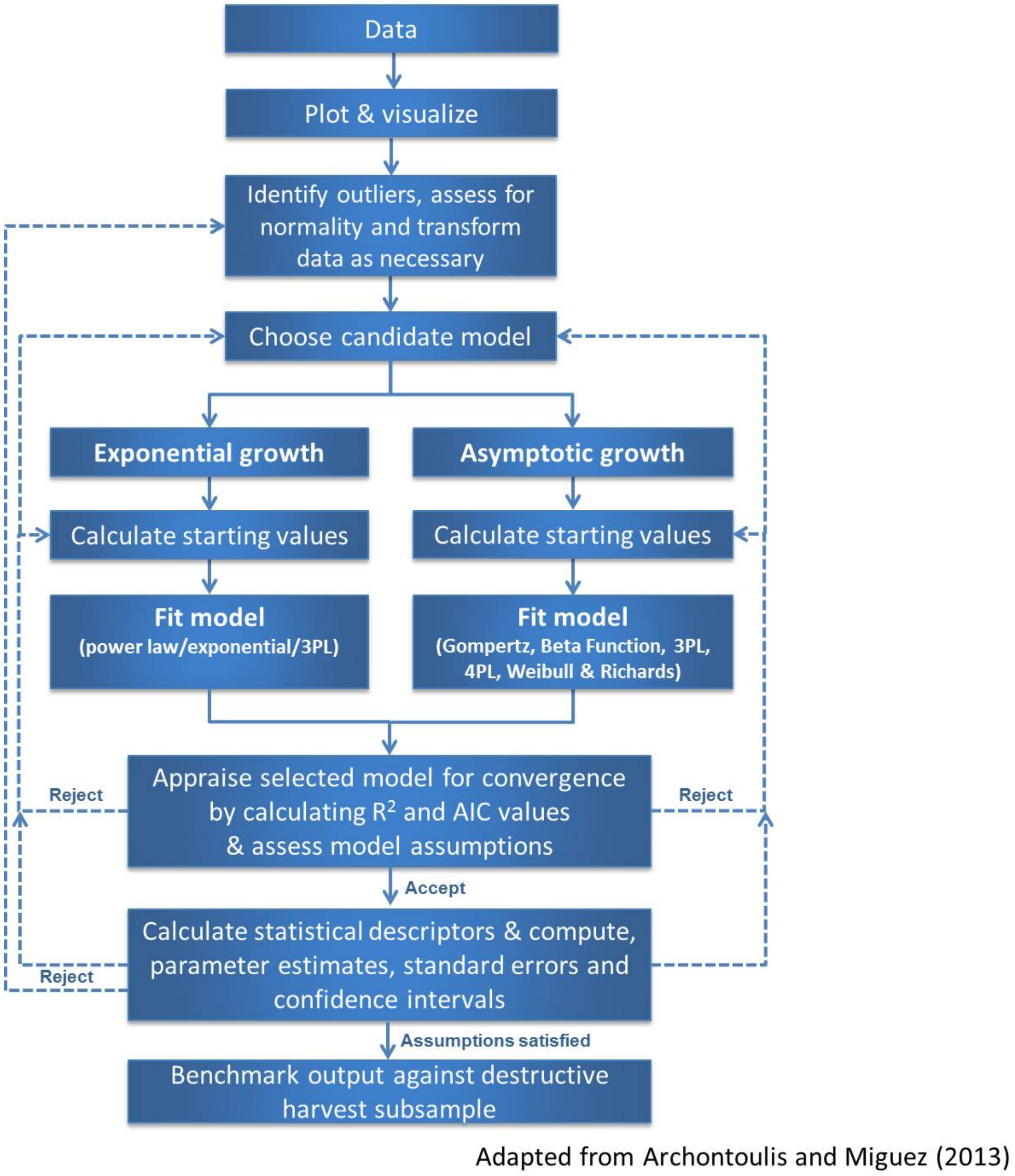

Supplementary Figure 1 Schematic overview for model selection and analysis (Abbreviations: 3PL, three parameter logistic; 4PL, four parameter logistic; AIC, Akaike information criterion). The models chosen for analysis are underlined.

Archontoulis SV, Miguez FE. 2013. Nonlinear Regression Models and Applications in Agricultural Research. *Agronomy Journal* **105**, 1-13. Reprinted by Permission, ASA, CSSA, SSSA.

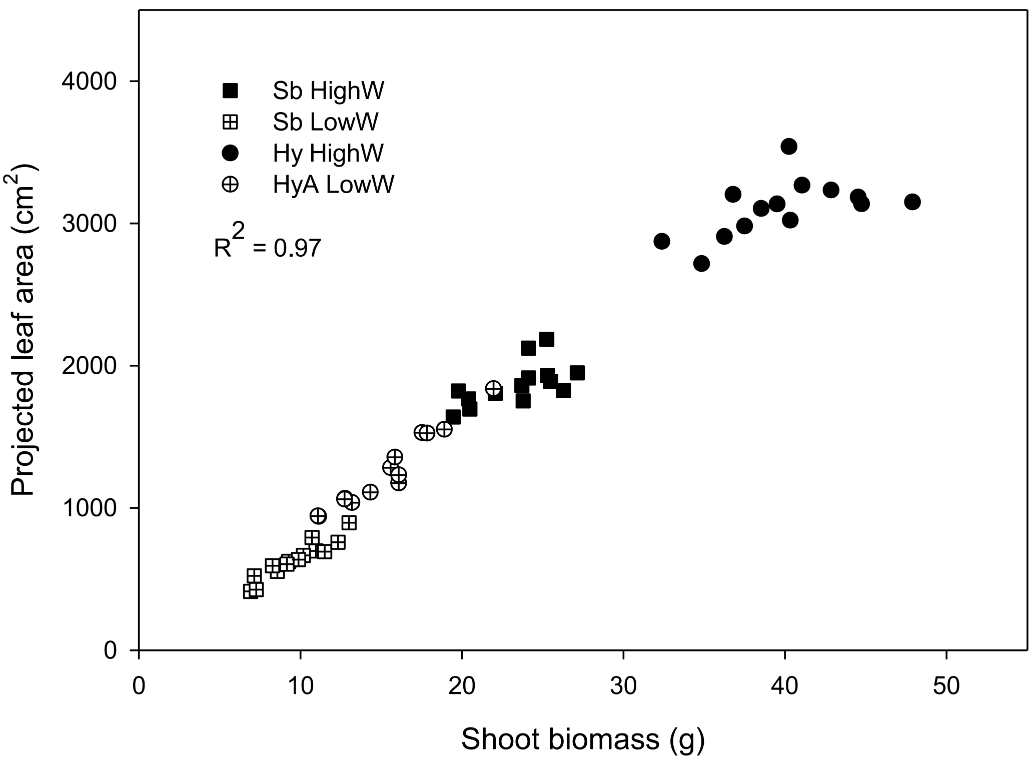

Supplementary Figure 2 Relationship between shoot biomass and projected leaf area calculated from image analysis for Sb and HyA plants subject to high and low water conditions. Projected leaf area provides a strong indication of plant growth with a significant, positive correlation observed ($P < 0.0001$; $R^2 = 0.97$; $y = 78.0x – 2.71$).

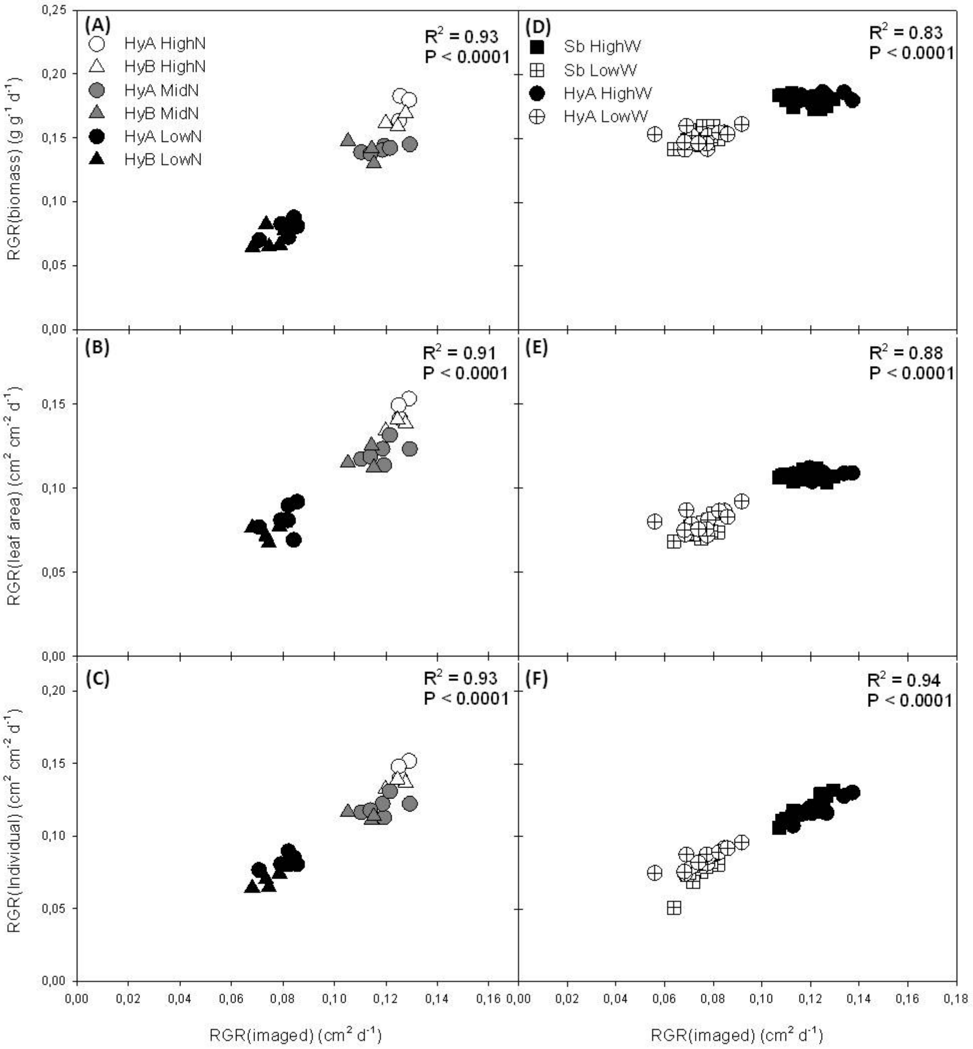

Supplementary Figure 3 Assessment of Relative Growth Rate (RGR) derived from the power law and 3PL models selected for the nutrient (A-C) and water limiting experiments (D-F), respectively. RGR(imaged) was derived from the models using continuous data, compared to RGR(biomass) calculated from initial biomass of a sample of plants and final biomass of replicate plants (A and D); RGR(area) was calculated using the final leaf area of individual plants and the pooled mean initial leaf area (B and E); and RGR(individual) was where both the initial and final leaf area of individual plants were used to calculate the growth. A strong correlation was detected between RGR(biomass) and RGR(imaged) when plants grown under different nitrogen levels (A, B; R^2 = 0.93 and 0.91, respectively) but not when plants were grown under water-limited conditions (D, E; R^2 = 0.83 and 0.88, respectively). A very high correlation was detected in both nutrient and water limiting experiments when the initial leaf area for each individual plant was used to calculate RGR (C, D; R^2=0.93 and 94, respectively).

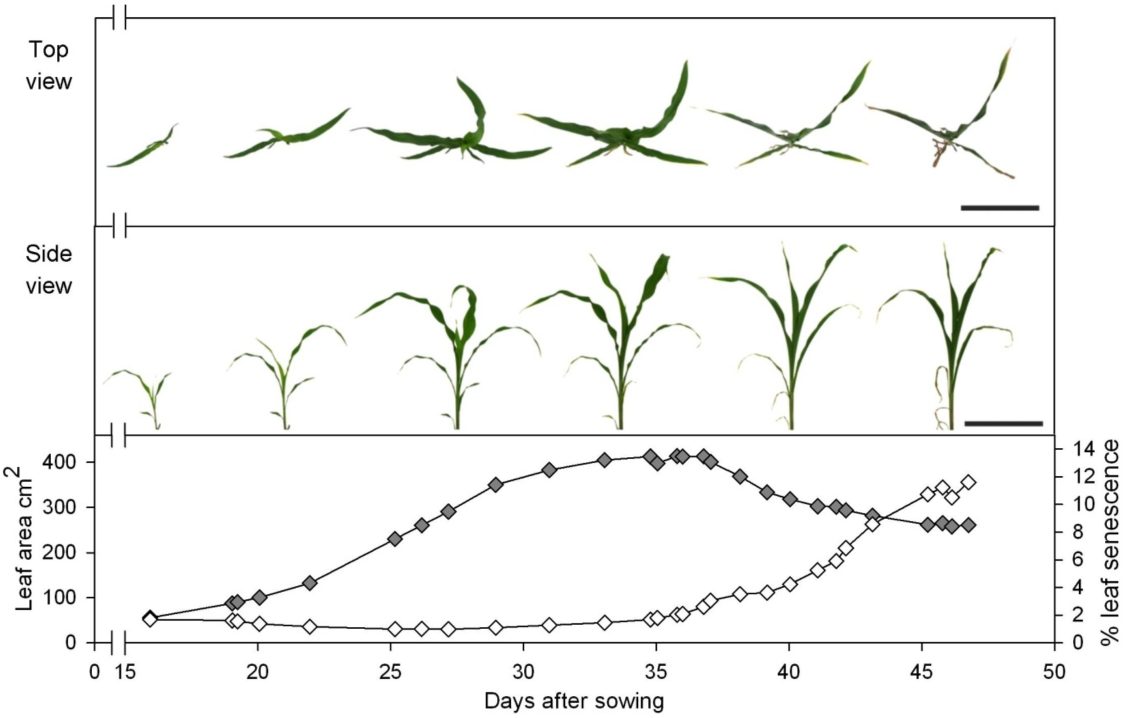

Supplementary Figure 4. Phenotypic response in a representative *Sorghum bicolor* (Sb) plant subjected to limited watering over four weeks as observed in images taken from above (A) and from the side (B). Leaf area (grey symbols, C) initially increased but then decreased as a result of leaf curling and senescence (white symbols).

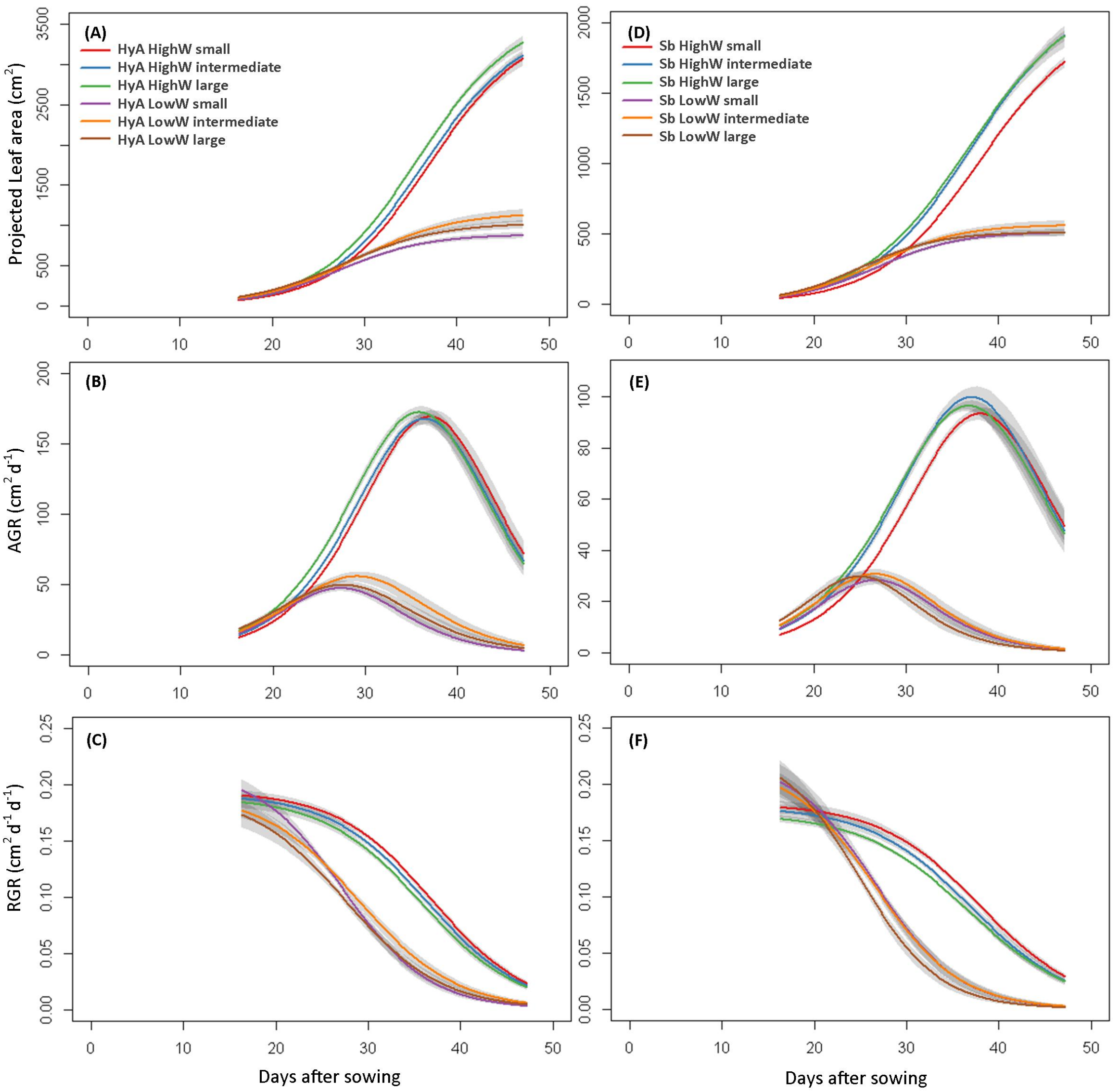

Supplementary Figure 5: Effect of seedling size (small, intermediate and large) on HyA (A-C) and Sb (D-F) growth under high and low watering regimes. Calculated projected leaf area (A, D), absolute growth rate (B, E) and relative growth rate (C, F) for the asymptotic 'water-limited' dataset fitted with the three parameter logistic model.

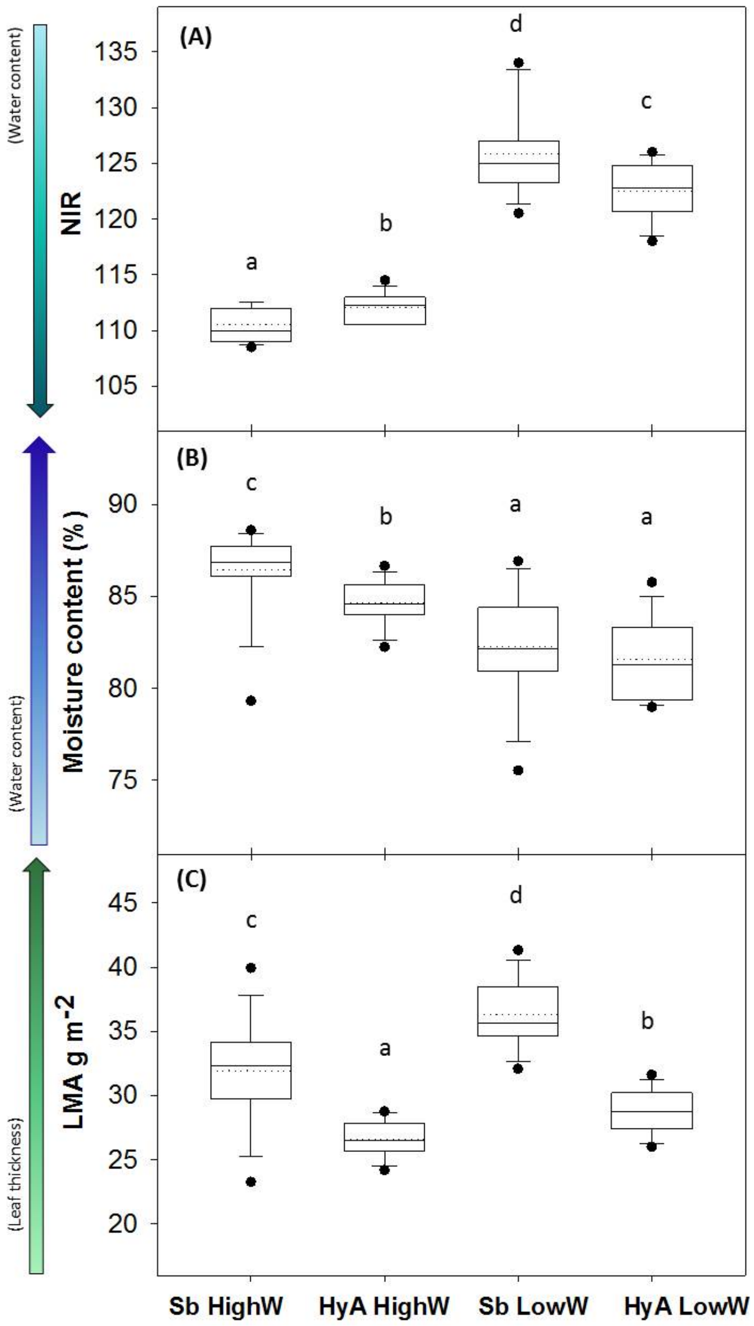

Supplementary Figure 6: Differences in NIR reflectance (A), moisture content (B) and LMA (leaf mass per area) (C) in Sb and HyA plants subjected to well watered and water limiting conditions. The significant differences in NIR (A) could not be accounted for by moisture content alone, as demonstrated by the similarity in moisture content observed between the two sorghum lines under low water conditions (B). However, a significant difference was observed, in LMA, which is used as a proxy for leaf thickness (C).

SUPPEMENTARY TABLES

Table S1

Summary of the two experiments including cultivars and the dates of sowing and imaging. The first experiment was a nitrogen trial using two different forage sorghum hybrids (HyA and Sweet Jumbo -LPA). The second experiment was a water supply trial using one forage sorghum hybrid (HyA) and a variety of grain sorghum (*Sorghum bicolor*; Sb).

Experiment	Genotypes	Date of sowing	Start imaging	End imaging
Expt1: Nitrogen	HyA and HyB	22/2/11	3/3/11	4/4/11
Expt 2: Water-limiting	HyA and Sb	23/12/11	8/1/12	8/2/12

Table S2

Summary of plant traits derived using LemnaGrid software using phenotypic imaging recorded in The Plant Accelerator®.

Trait	Description
NIR	Near Infrared Reflectance. Images taken at 750-1400 nm, and used as a measure of water content.
Senescence (%)	Measure of senescent (yellow) tissue using the relative proportion of pixels in the RGB images made using visible light.
Mean HUE angle	Measure of plant "greenness", estimated by the conversion of RGB to HSI colour.
Compactness	Ratio of leaf area per convex hull area calculated from the top view image. Trait may be a proxy of leaf area index.
Convex Hull Area (cm^2)	The area within the smallest mathematically solved perimeter that envelopes the plant when imaged from above.
Eccentricity	Mathematical measure of how a conic section deviates from being circular from the top view image. This trait may be used as a proxy for plant symmetry, such that the closer the value is to 0, the more radially symmetrical the plant is.
Caliper Length (cm)	The longest line that can be drawn between the pixels within the convex hull from the top view image. This trait may be used as a measure of how wide and how far spread the leaves are.
Surface Coverage	Ratio of leaf area per smallest mathematically solved circle area enclosing the plant calculated from the top view image. Trait may be a proxy for leaf area index.

Table S3 (A)

Growth curve parameters for the modelled projected shoot area vs days after sowing for the nitrogen experiment with the calculated R^2 values (See equation 2 and equation 3 in the Materials and Methods of the main text). Two varieties of forage sorghum (HyA and HyB) were grown at three different levels of nitrogen for 4 weeks and imaged daily.

Model	Parameter	HyA HighN	HyA MidN	HyA LowN	HyB HighN	HyB MidN	HyB LowN
**Exponential**	M_0	2.5 ± 0.3	2.2 ± 0.1	2.1 ± 0.1	No convergence	3.1 ± 0.3	2.5 ± 0.3
	r	0.13 ± 0.004	0.12 ± 0.002	0.08 ± 0.002		0.11 ± 0.003	0.07 ± 0.003
	R^2	0.948	0.921	0.907		0.932	0.847
	AIC	541	895	583		804	544
**Power Law**	r	0.21 ± 0.01	0.20 ± 0.004	0.15 ± 0.01	0.25 ± 0.01	0.22 ± 0.01	0.16 ± 0.01
	beta	0.90 ± 0.01	0.88 ± 0.01	0.81 ± 0.02	0.87 ± 0.01	0.85 ± 0.01	0.75 ± 0.03
	R^2	0.963	0.974	0.916	0.992	0.954	0.824
	AIC	522	836	570	479	771	544
**3-parameter Logistic**	Asym	1000 ± 130	620 ± 30	140 ± 20	1130 ± 80	600 ± 70	600 ± 1500
	t_{mid}	40 ± 1	40 ± 1	43 ± 3	40 ± 1	40 ± 2	70 ± 43
	k	5.6 ± 0.2	6.3 ± 0.1	9.2 ± 0.6	5.9 ± 0.2	6.6 ± 0.3	12.9 ± 1. 9
	R^2	0.948	0.985	0.916	0.985	0.951	0.843
	AIC	511	786	571	463	763	546

Table S3 (B)

Growth curve parameters for the modelled projected shoot area vs days after sowing for the watering experiment with the calculated R^2 values (See equation 2 and equation 3 in the Materials and Methods of the main text).). Two varieties of sorghum, a hybrid (HyA) and a variety of *Sorghum bicolor* (Sb) were grown for 4 weeks and either well-watered (High W) or watered to 25% of field capacity (Low W).

Model	Parameter	Sb HighW	Sb LowW	HyA HighW	HyA LowW
**3-parameter Logistic**	Asym	2160 ± 40	520 ± 90	3500 ± 50	1020 ± 20
	t_{mid}	37.4 ± 0.3	25.9 ± 0.3	36.2 ± 0.2	28.1 ± 0.3
	K	5.62 ± 0.08	4.38 ± 0.12	5.17 ± 0.06	5.08 ± 0.13
	R^2	0.968	0.728	0.978	0.720
	AIC	4592	4435	4881	4915
**Gompertz**	Asym	4500 ± 300	590 ± 20	6200 ± 300	1320 ± 60
	k	12.0 ± 0.3	16.2 ± 1.3	14.3 ± 0.5	11.0 ± 0.6
	r	0.945 ± 0.002	0.892 ± 0.004	0.936 ± 0.002	0.918 ± 0.003
	R^2	0.965	0.729	0.970	0.759
	AIC	4638	4477	5046	4887
**Beta Function**	w_{max}	1920 ± 50	492 ± 7	No Convergence	1030 ± 20
	t_e	49.7 ± 0.6	35.8 ± 0.5		43.8 ± 0.8
	t_m	39.4 ± 0.4	27.8 ± 0.2		31.8 ± 0.4
	R^2	0.967	0.716		0.748
	AIC	4614	4467		4933
**4-parameter Logistic**	M_{min}	-7.1 ± 7.5	No Convergence	23 ± 8	No Convergence
	M_{max}	2200 ± 60		3400 ± 60	
	t_{mid}	37.6 ± 0.4		35.9 ± 0.2	
	k	5.8 ± 0.2		4.9± 0.1	
	R^2	0.968		0.979	
	AIC	4592		4877	
**Richards**	M_{max}	2260 ± 100	No Convergence	3250 ± 70	No Convergence
	t_{mid}	37 ± 1		37.5 ± 0.3	
	k	6.3 ± 0.7		3.9 ± 0.3	
	l_{pow}	-0.17 ± 0.18		0.4 ± 0.1	
	R^2	0.968		0.979	
	AIC	4592		4873	
**Weibull**	M_{max}	2060 ± 60	57 ± 2	3170 ± 50	No Convergence
	drop	2030 ± 70	-508 ± 17	3110 ± 50	
	lrc	-18.2 ± 0.4	13.1 ± 0.7	-20.4 ± 0.4	
	p	4.9 ± 0.1	-4.1 ± 0.2	5.6 ± 0.1	
	R^2	0.968	0.731	0.979	
	AIC	4592	4413	4877	

Table S3 (C)

Equations for eight models used to assess the growth of sorghum in two sets of data obtained from The Plant Accelerator ®. Growth models can be classified under two broad categories, those that assume no asymptotic final size exists (e.g. exponential growth) and those that do assume one exists (eg logistic curve). Non-Asymptotic growth models make the assumption that growth will continue indefinitely. Although this assumption may be unrealistic for plants through the entire lifetime of a plant, they may be appropriate for modelling the growth of young seedlings. The ability of each model to describe the data was assessed using convergence and correlative analysis (see Table S3A and S3B).

Exponential

The exponential model implies that the rate of change of a measured quantity is proportional to the measured quantity.

$M(t) = M_0 e^{rt}$ Equation A.1

$AGR = \frac{dM}{dt} = M_0 r e^{rt}$ Equation A.2

$RGR = \frac{1}{M}\frac{dM}{dt} = r$ Equation A.3

Where
M is the measured value
t is the time after sowing
M_0 is the initial measured value at time t_0
r is a parameter controlling the relative growth rate.

Power Law

The power law model is a non-asymptotic model that allows both AGR and RGR to vary throughout time. 0 > β > 1 implies a progressive decrease in Relative growth rate. The linear model is obtained when β=0 and the model converges to the exponential model as β approaches 1.

$M(t) = \left({M_0}^{1-\beta} + rt(1-\beta)\right)^{\frac{1}{1-\beta}}$ Equation B.1

$AGR = \frac{dM}{dt} = r\left({M_0}^{1-\beta} + rt(1-\beta)\right)^{\frac{\beta}{1-\beta}}$ Equation B.2

$RGR = \frac{1}{M}\frac{dM}{dt} = \frac{r}{\left({M_0}^{1-\beta} + rt(1-\beta)\right)}$ Equation B.3

Where
M is the measured value
t is the time after sowing
M_0 is the initial measured value at time t_0
r is a parameter controlling the growth rate.
β controls the progressive changes to RGR.

3-paramater logistic

The 3-parameter logistic model is the most commonly used asymptotic model used to model growth. The lower asymptote of the 3 parameter version of the logistic curve is fixed at 0. Although this may not be strictly realistic as there is an inherit seed weight associated with a plant, this is generally small enough to be ignored for the purpose of curve fitting. In addition the inflection point of the curve (point at which the AGR is maximised) falls at half the expected upper asymptotic value. Other versions of the logistic curve can relax one or both of these constraints. The 3-parameter logistic function is implemented in R with the *SSlogistic* function in the nlme package.

$$M(t) = \frac{M_{max}}{1+e^{\left(\frac{t_{mid}-t}{k}\right)}}$$ Equation C.1

$$AGR = \frac{dM}{dt} = \frac{M_{max}e^{\frac{t_{mid}+t}{k}}}{k\left(e^{\frac{t_{mid}}{k}}+e^{\frac{t}{k}}\right)^2}$$ Equation C.2

$$RGR = \frac{1}{M}\frac{dM}{dt} = \frac{e^{\frac{t_{mid}+t}{k}}\left(1+e^{\frac{t_{mid}-t}{k}}\right)}{k\left(e^{\frac{t_{mid}}{k}}+e^{\frac{t}{k}}\right)^2}$$ Equation C.3

Where
M is the measured value
t is the time after sowing
M_{max} is the upper asymptote
t_{mid} represents the inflection point of the model
k is a parameter controlling the maximal growth rate.

Gompertz

The gompertz model allows RGR to fall exponentially over time. This differs from the logistic model in that the time of maximal growth appears at approximately 37% of the final expected biomass. Accordingly, the end result of the upper asymptote is approached more slowly than the lower asymptote. Like the 3 parameter logistic model, the gompertz model assumes an initial measured value of 0. The gompertz model is implemented in R with the *SSgompertz* function in the nlme package.

$$M(t) = Asym\ e^{-kr^t}$$ Equation D.1

$$AGR = \frac{dM}{dt} = k\ Asym\ r^t\left(-e^{-kr^t}\right)\log_e r$$ Equation D.2

$$RGR = \frac{1}{M}\frac{dM}{dt} = -kr^t \log_e r$$ Equation D.3

Where
M is the measured value
t is the time after sowing
Asym is the upper asymptote
k displaces the graph along the y axis
r controls the maximal growth rate

Beta Function

The beta function is a 3 parameter sigmoidal function that allows flexibility in the time of maximal AGR. It does this using only 3 parameters allowing for easier convergence. However the model is not continuous (Yin *et al*, 2003).

$$M(t) = \begin{cases} 0, & t < 0 \\ M_{max}\left(1 + \frac{t_{end}-t}{t_{end}-t_{mid}}\right)\left(\frac{t}{t_{end}}\right)^{\frac{t_{end}}{(t_{end}-t_{mid})}}, & 0 \le t \le t_{end} \\ M_{max}, & t > t_{end} \end{cases} \quad \text{Equation E.1}$$

$$AGR = \frac{dM}{dt} = \begin{cases} 0, & t < 0 \\ c_m\left(\frac{t_{end}-t}{t_{end}-t_{mid}}\right)\left(\frac{t}{t_{mid}}\right)^{\frac{t_{mid}}{t_{end}-t_{mid}}}, & 0 \le t \le t_{end} \\ 0, & t > t_{end} \end{cases} \quad \text{Equation E.2}$$

Where

$$c_m = M_{max}\left(\frac{2t_{end}-t_{mid}}{t_{end}(t_{end}-t_{mid})}\right)\left(\frac{t_{mid}}{t_{end}}\right)^{\frac{t_{mid}}{t_{end}-t_{mid}}}$$

$$RGR = \frac{1}{M}\frac{dM}{dt} = \frac{(2t_{end}-t_{mid})(t_{end}-t)}{(t_{end}-t_{mid})(2t_{end}-t_{mid}-t)t} \quad \text{Equation E.3}$$

Where
M is the measured value
t is the time after sowing
M_{max} is the maximal measured value
t_{end} is the time that M_{max} is achieved
t_{mid} represents the inflection point of the model

4-paramater Logistic

The 4-parameter version of the logistic function relaxes the constraint on the lower asymptote, allowing non-zero values. The 3-parameter logistic model is obtained when M_{min} equals 0. The four parameter logistic function is implemented in R with the *SSfpl* function in the nlme package.

$$M(t) = \frac{M_{min}(M_{max}-M_{min})}{1+e^{\left(\frac{t_{mid}-t}{k}\right)}} \quad \text{Equation F.1}$$

$$AGR = \frac{dM}{dt} = \frac{M_{min}(M_{max}-M_{min})e^{\frac{t_{mid}-t}{k}}}{k\left(e^{\frac{t_{mid}-t}{k}}+1\right)^2} \quad \text{Equation F.2}$$

$$RGR = \frac{1}{M}\frac{dM}{dt} = \frac{1}{k\left(e^{\frac{t-t_{mid}}{k}}+1\right)} \quad \text{Equation F.3}$$

Where
M is the measured value
t is the time after sowing
M_{min} is the lower asymptote
M_{max} is the upper asymptote
t_{mid} represents the inflection point of the model

k is a parameter controlling the maximal growth rate.

Weibull

$$M(t) = \frac{M_{max} - drop}{e^{e^r t^p}} \qquad \text{Equation G.1}$$

$$AGR = \frac{dM}{dt} = p(drop - M)t^{p-1}e^{r-e^r t^p} \qquad \text{Equation G.2}$$

$$RGR = \frac{1}{M}\frac{dM}{dt} = p(-e^r)t^{p-1} \qquad \text{Equation G.3}$$

Where
M is the measured value
t is the time after sowing
M_{max} is the upper asymptote
drop represents the change from M_{max} to the y intercept
r controls the maximal growth rate
p is a numerical constant representing the power to which x is raised

Richards

The Richards curve is a 4 parameter version of the logistic function that relaxes the constraint on the location of maximal growth. The 3-parameter logistic model is obtained when p equals 0. The Richards function is implemented in R with the *SSRichards* function in the NRAIA package.

$$M(t) = \frac{M_{max}}{\left(1+e^{\frac{t_{mid}-t}{k}}\right)^{e^{-p}}} \qquad \text{Equation H.1}$$

$$AGR = M_{max}e^{-\frac{kp-t_{mid}-t}{k}}\left(e^{\frac{t_{mid}-t}{k}} + 1\right)^{-e^{-p}-1} \qquad \text{Equation H.2}$$

M is the measured value
t is the time after sowing
M_{min} is the lower asymptote
M_{max} is the upper asymptote
t_{mid} represents the inflection point of the model
k is a parameter controlling the maximal growth rate.
p controls the location of maximal growth.

Table S4

R scripts used to calculate the fitted growth models, absolute growth rates (AGR) and relative growth rates (RGR) along with the 95% confidence intervals for sorghum varieties used in the nitrogen and water-limiting experiments. Models are adapted from (Paine *et al.*, 2012). Text beginning with '#' contains comments explaining the function of the code following the statement, how to use the function and what it produces. The main driver of the script (output.growth.model) also shows a brief "how to use" with example data that is included in R.

```
# Summarizer function which takes the data set and an alpha value and
returns
# the high and low value for each data point within the confidence interval
specified
# in alpha (Adapted from Paine et al 2012)
#
# Example usage
# dataMatrix <- matrix(c(0,1,2,3,4,5,6,7,8,9,10))
# summarizer(list(growth=dataMatrix), 0.05)
#
# Expected Result:
# growth.lo   growth.hi
# 0.5         9.5
summarizer <- function(dat, alpha){
  n          <- length(dat)
  quantiles  <- c(alpha/2, 1-(alpha/2))
  CIs        <- data.frame(matrix(NA, ncol(dat[[1]]), n*2))
  names(CIs) <- paste(rep(names(dat), each = 2), c("lo", "hi"), sep = ".")

  for(i in 1:n){
    CIs[,(2*i-1):(2*i)] <- t(apply(dat[[i]], 2, quantile, quantiles, na.rm
= T))
  }

  return(CIs)
}
```

```
# General function to generate output data for models (Adapted from Paine et al 2012)

# This cfunction can be used to produce values for the model provided, its first

# derivitive (Absolute growth rate) and the first derivitive devided by the model

# (Relative growth rate) and optionally the confidence intervals around the model.

#

# The function takes any nls or gnls object (from the 'nlme' R package). The function

# also requires the underlying model function to generate the first derivitive of the

# model. The function will also optionally calculate the confidence intervals for the

# growth model, the AGR and the RGR with the provided alpha value. (Adapted from Paine et al 2012)

#

# Parameters:

#  fit: a nls or gnls fitted model (from the nlme package)

#  times: a sequence of time points to calculate

#  formula: an expression containing the formula for the model used to fit the data

#  indVar: string representation of the indepedant variable used in the formula provided (defaults to 'x')

#  CI: Boolean value determining whether or not to calculate the confidence intervals (defaults to False)

#  alpha: the alpha cut-off for confidence intervals (defaults to 0.05)

#

# Output:

#  params: A list of the calculated parameters for the fitted model

#  stats: A list containing the R^2, AIC and RMSE values

#  data: The fitted and rediduals calculated from the model

#  rates: The M (model value), AGR, RGR and optionally the high and low confidence intervals

#        for the M, AGR and RGR for each of the time points provided
```

```
#  summary: Summary statistics including:
#                    AvAGR: Average AGR
#                    AvRGR: Average RGR
#                    PeekTime: The time of maximal absolute growth rate
#                    PeekAGR: The maximal absolute growth rate
#                    PeekTime.hi: The high confidence interval for the time of maximal absolute growth rate
#                    PeekTime.lo: The low confidence interval for the time of maximal absolute growth rate
#                    PeekAGR.hi: The high confidence interval for maximal absolute growth rate
#                    PeekAGR.lo: The low confidence interval for maximal absolute growth rate
#
# Example usage (fitting the R default ChickWeight dataset to the 3 parameter logistic model)
# library(nlme)
# logisticFit <- gnls(weight ~ SSlogis(Time, Asym, xmid, scal), ChickWeight, weights=varPower())
# logisticFit <- gnls(weight ~ SSlogis(Time, Asym, xmid, scal), ChickWeight, weights=varPower())
# formula     <- expression(Asym/(1+exp((xmid-x)/scal)))
# times       <- seq(1, 50,by = 0.1)
# model       <- output.growth.model(logisticFit, times, formula, indVar='x', CI=T, alpha=0.05)
#
library(mvtnorm)
output.growth.model <- function(fit, times, formula, indVar='x', CI=F, alpha=0.05){
  param <- coef(fit)

  #
  if(inherits(fit, "gnls")){
    fitted <-fit$fitted
    resid  <- fit$resid
  }else{ #Assume nls
```

```
  fitted <- fit$m$fitted()
  resid  <- fit$m$resid()
}

data <- data.frame(fitted = fitted, resid = resid)
mss <- sum((fitted - mean(fitted))^2)
rss <- sum(resid^2)
R2  <- mss/(mss + rss)
rmse <- sqrt(rss)
AIC <- AIC(fit)

assign(toString(indVar), times)
AGRFormula <- D(formula,toString(indVar))
rates = data.frame(
  times = times,
  M    = eval(formula, as.list(param)),
  AGR  = eval(AGRFormula, as.list(param))
)
rates$RGR = rates$AGR/rates$M

peekAGRm <- max(rates$AGR)
peekAGRt <- rates$times[which(rates$AGR==max(rates$AGR))]
avAGR <- mean(rates$AGR)
avRGR <- mean(rates$RGR)

if(CI == T){
  if(inherits(fit, "gnls")){
    cov   <- fit$varBeta
  }else{
    cov   <- summary(fit)$cov
  }
  .x <- data.frame(rmvnorm(n=1000, mean=param, sigma=cov))
  M <- AGR <- RGR <- matrix(NA, ncol = length(times), nrow = nrow(.x))
  peakAGRt <- c()
```

```
    peakAGRm <- c()
    for(i in 1:nrow(.x)){
      M[i,]     <- eval(formula, as.list(.x[i,]))
      AGR[i, ]  <- eval(AGRFormula, as.list(.x[i,]))
      RGR[i,] <- AGR[i,]/M[i,]

      peakAGRm[i] <- max(AGR[i, ])
      peakAGRt[i] <- rates$times[which(AGR[i, ]==max(AGR[i, ]))]
    }

    CIs <- summarizer(list(M = M, AGR = AGR, RGR = RGR), alpha)
    rates = cbind(rates, CIs)

    out <- list(params = param, stats = c(R2 = R2, AIC = AIC, RMSE = rmse),
                data = data, rates = rates,
                summary=c(AvAGR=avAGR, AvRGR=avRGR,
                          PeekTime=peekAGRt, PeekAGR=peekAGRm,
                          PeekTime.hi = quantile(peakAGRt, 1-alpha/2, na.rm
= T)[1],
                          PeekTime.lo = quantile(peakAGRt, alpha/2, na.rm =
T)[1],
                          PeekAGR.hi = quantile(peakAGRm, 1-alpha/2, na.rm
= T)[1],
                          PeekAGR.lo = quantile(peakAGRm, alpha/2, na.rm =
T)[1]
                ))

  }else{

    out <- list(params = param, stats = c(R2 = R2, AIC = AIC, RMSE = rmse),
                data = data, rates = rates,
                summary=c(AvAGR=avAGR, AvRGR=avRGR,
                          PeekTime=peekAGRt, PeekAGR=peekAGRm))
  }
}
```

Table S5: Chlorophyll (Chl), HCN (dhurrin), nitrate (NO_3^-) concentration, and total nitrogen allocation in the leaf tissue of two hybrid sorghum varieties grown under different nitrogen conditions. Mean ± standard error and significance of a two-way ANOVA are presented for each variety (V) and treatment (T).

	**Hybrid A**			**Hybrid B**			**Statistics**		
	**High N**	**Mid N**	**Low N**	**High N**	**Mid N**	**Low N**	**V**	**T**	**V×T**
Total N (%)	4.7 ± 0.1	2.0 ± 0.1	1.2 ± 0.03	4.7 ± 0.1	1.9 ± 0.1	1.2 ± 0.1	ns	***	ns
CN^- mg g^{-1}	0.5 ± 0.1	0.5 ± 0.03	0.1 ± 0.02	0.5 ± 0.04	0.4 ± 0.1	0.1 ± 0.03	ns	***	ns
NO_3^- mg g^{-1}	11.7 ± 0.5	4.8 ± 0.5	4.7 ± 0.6	10.7 ± 0.5	3.9 ± 0.3	4.4 ± 0.3	ns	***	ns
Chl mg g^{-1}	7.3 ± 0.3	6.9 ± 0.2	4.2 ± 0.2	7.7 ± 0.4	6.7 ± 0.2	5.0 ± 0.2	ns	***	ns

*P< 0.05; **P< 0.01; ***P< 0.001; ns, not significant

Table S6: Chlorophyll (Chl), HCN (dhurrin) and nitrate (NO_3^-) concentration, and total nitrogen allocation in the leaf tissue of well-watered and drought stressed *Sorghum bicolor* and forage sorghum HyA. Mean ± standard error and significance of a two-way ANOVA are presented for each variety (V) and treatment (T).

	S. bicolor		**HyA**		**Two-way ANOVA**		
	**WW**	**WL**	**WW**	**WL**	**V**	**T**	**V ×T**
Total N (%)	2.7 ± 0.1	3.6 ± 0.1	2.4 ± 0.1	3.1 ± 0.1	***	***	ns
CN^- (mg g^{-1})	0.3 ± 0.1	0.9 ± 0.1	0.5 ± 0.1	0.9 ± 0.1	ns	***	ns
NO_3^- (mg g-1)	6.6 ± 0.7	16.2 ± 1.5	2.5 ± 0.1	9.9 ± 0.3	***	***	ns
Chl (mg g^{-1})	8.4 ± 3.4	7.5 ± 3.1	7.2 ± 2.9	7.8 ± 3.2	ns	ns	ns

WW – well watered; WL – water limited. *P< 0.05; **P< 0.01; ***P< 0.001; ns, not significant
